# Isolation, identification, and characterization of novel nanovesicles

**DOI:** 10.18632/oncotarget.9325

**Published:** 2016-05-12

**Authors:** Huang-Ge Zhang, Pengxiao Cao, Yun Teng, Xin Hu, Qilong Wang, Ashish S. Yeri, Xiaoying Zhuang, Abhilash Samykutty, Jingyao Mu, Zhong-Bin Deng, Lifeng Zhang, James A. Mobley, Jun Yan, Kendall Van Keuren-Jensen, Donald Miller

**Affiliations:** ^1^ Louisville Veterans Administration Medical Center, Louisville, KY 40206, USA; ^2^ James Brown Cancer Center, Department of Microbiology and Immunology, University of Louisville, KY 40202, USA; ^3^ Department of Medicine, University of Louisville, KY 40202, USA; ^4^ Program in Biostatistics, Bioinformatics and Systems Biology, The University of Texas Graduate School of Biomedical Sciences at Houston, TX 77030, USA; ^5^ Department of Genomic Medicine, The University of Texas MD Anderson Cancer Center, Houston, TX 77030, USA; ^6^ Translational Genomics Research Institute, Phoenix, AZ 85004, USA; ^7^ Mass Spectrometry/Proteomics Shared Facility, University of Alabama at Birmingham, Birmingham, AL 35294, USA; ^8^ Department of Clinical Oncology, Huai'an First People's Hospital, Nanjing Medical University, Huai'an, 223300, China

**Keywords:** isolation and identification extracellular microvesicles, in vivo predominately population, HG-NV, exosomes

## Abstract

Extracellular microvesicles (EVs) have been recognized for many potential clinical applications including biomarkers for disease diagnosis. In this study, we identified a major population of EVs by simply screening fluid samples with a nanosizer. Unlike other EVs, this extracellular nanovesicle (named HG-NV, HG-NV stands for HomoGenous nanovesicle as well as for Huang-Ge- nanovesicle) can be detected with a nanosizer with minimal *in vitro* manipulation and are much more homogenous in size (8–12 nm) than other EVs. A simple filtration platform is capable of separating HG-NVs from peripheral blood or cell culture supernatants. In comparison with corresponding exosome profiles, HG-NVs released from both mouse and human breast tumor cells are enriched with RNAs. Tumor derived HG-NVs are more potent in promoting tumor progression than exosomes. In summary, we identified a major subset of EVs as a previously unrecognized nanovesicle. Tumor cell derived HG-NVs promote tumor progression. Molecules predominantly present in breast tumor HG-NVs have been identified and characterized. This discovery may have implications in advancing both microvesicle biology research and clinical management including potential used as a biomarker.

## INTRODUCTION

Intercellular communication is a hallmark of multicellular organisms. Recently, extracellular microvesicles (EVs) have been recognized as one of the major mechanisms for intercellular communication [1–3]. EVs have been isolated from diverse body fluids, including semen [4], blood [5], urine [6], saliva [7], breast milk [8], amniotic fluid [9], ascites fluid [10, 11], cerebrospinal fluid [12–16], and bile [17, 18]. However, EVs include more than one type, and whether a particular subpopulation of EVs is the predominant type in a specimen or upon isolation is not known.

The recent increase of EV research has strongly emphasized the application of these nanovesicles as diagnostic and treatment monitoring tools [19–22]. Utilizing the most abundant EVs circulated in the body fluid will be the best resource for such applications. A primary class of EVs is thought to be exosomes. However, current protocols used for isolation of exosomes [3], do not aid in determining if exosomes are the most abundant EVs in a sample. Specifically, whether the presence of other types of vesicles from exosome-depleted supernatants is overlooked and has not been investigated. Moreover, exosomes carry various proteins, bioactive lipids and genetic information to alter the phenotype and function of recipient cells. Thus, exosomes have been implicated in numerous biological and pathological processes [3, 23, 24]. Like other EVs, exosomes are heterogeneous in size (50–150 nm) and in function, and are released from many cell types. The heterogeneity of exosomes makes it very challenging to determine if a specific subpopulation of exosomes is the dominate subpopulation or phenotype in a clinical specimen. Current strategies for charactering exosomes are limited to multiple *in vitro* manipulations for isolation and purification, followed by analytic approaches that generate data that may not represent what takes place *in vivo*. Therefore, the ability to identify, isolate, and molecularly characterize EVs with minimal *in vitro* manipulation is urgently needed. In this study, we demonstrated that unlike other identified EVs including exosomes which cannot be detected using a nanosizer without concentration *in vitro*, HG-NVs which are 8–12 nm in size can be readily detected from blood and cell cultured supernatants without *in vitro* manipulations. As proof of concept, HG-NVs released from mouse and human breast tumor cells were further characterized. HG-NVs have a number of unique characteristics in comparison with corresponding exosomes purified from identical samples. HG-NVs released from tumor cells are relatively homogenous in size; have specific RNAs induced in a disease dependent manner in a mouse breast tumor model and a LPS induced septic shock mouse model; and higher percentages of PS lipids. In combination with the feature that HG-NVs are a predominate set of EVs, HG-NVs could be utilized as a better source for disease diagnosis. The biological effect of HG-NVs on promoting tumor progression was further demonstrated in lung and liver metastasis of murine breast tumor and colon cancer models. Collectively, the identification of previously unrecognized HG-NVs may also increase our fundamental understanding of the biology of EVs and increase diagnostic value for a non-invasive diagnostic and screening tool to detect stages of certain types of cancers.

## RESULTS

**Figure 1:**
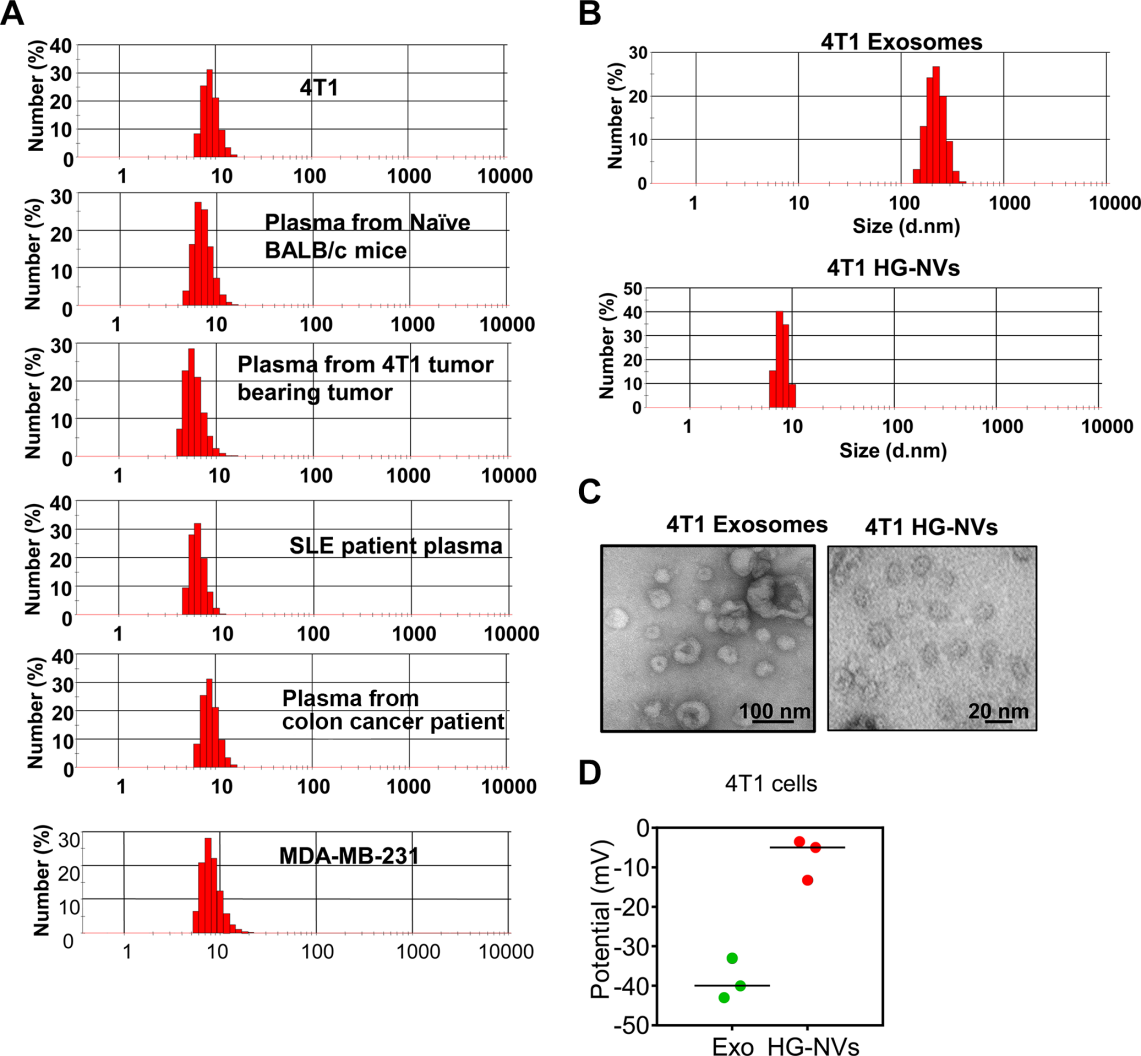
Identification and characterization of HG-NV (**A**) 800 μl of samples were added to the cuvette, and the size distribution was determined using the Zetasizer Nano ZS. (**B**) The size distribution of banded 4T1 samples from sucrose gradient ultracentrifugation was visualized by the Zetasizer Nano ZS (B) and electromicroscopy (**C**). The surface Zeta-potential of the particles was determined using the Zetasizer Nano ZS (**D**). Results (A–C) represent one of three independent experiments.

The heterogeneous size of EVs is based on data generated from EVs after multiple *in vitro* manipulations. The identification of EVs prior to isolation by *in vitro* manipulations was not possible. We first examined peripheral blood collected from naïve and tumor bearing mice, healthy subjects and diseased patients, and the cell culture supernatants using a standard nanosizer (Zetasizer Nano ZS). We observed that all samples we examined, predominantly contained nanosize particles (Figure 1A). Nanosize particles were detected in the blood of naïve mice (8.79 ± 1. 68 nm), 4T1 breast tumor bearing mice (7.12 ± 2.11 nm), SLE patients (7.69 ± 1.57 nm) and colon cancer patients (9.25 ± 1.37 nm), means ± S.E.M). Nanosized particles have also been detected in cell culture supernatants of 4T1 cells (9.41 ± 1.83 (nm) and of MDA-MB-231 human breast tumor cell line (8.94 ± 2.55 (nm) indicating that EVs with a diameter of 8–12 nm are readily detected in blood and cell culture supernatants. The presence of the EVs with a diameter of 8–12 nm are also observed in the blood samples of mice with acute inflammation induced by an IP injection of LPS and in blood samples from different genetic background mice (C57BL/6 versus BALB/c) (Supplementary Table 1). Therefore, unlike other EVs, with minimal *in vitro* manipulation this extracellular HG-NV can be detected with a nanosizer and are much less heterogeneous in size (8–12 nm) than other EVs (for an example, exosomes, 50–150 nm, microparticles 300–1,000 nm). To further characterize the HG-NVs released from 4T1 tumor cells, HG-NVs from exosome-depleted samples were isolated with a simple column infiltration method. The column filtration consists of a filter with 500 kDa cutoff (Supplementary Figure 1) and pumped to regulate the speed of fluid passing through the column. After a simple, one step procedure for sample concentration with the column infiltration, followed by sucrose gradient purification, the size distribution of the HG-NVs was determined using a nanosizer (Figure 1B) and confirmed by electron microscopy (Figure 1C). HG-NVs are less charged (Figure 1D) than exosomes isolated from the same sample used for HG-NV isolation.

### Identification of HG-NV RNA composition

**Figure 2:**
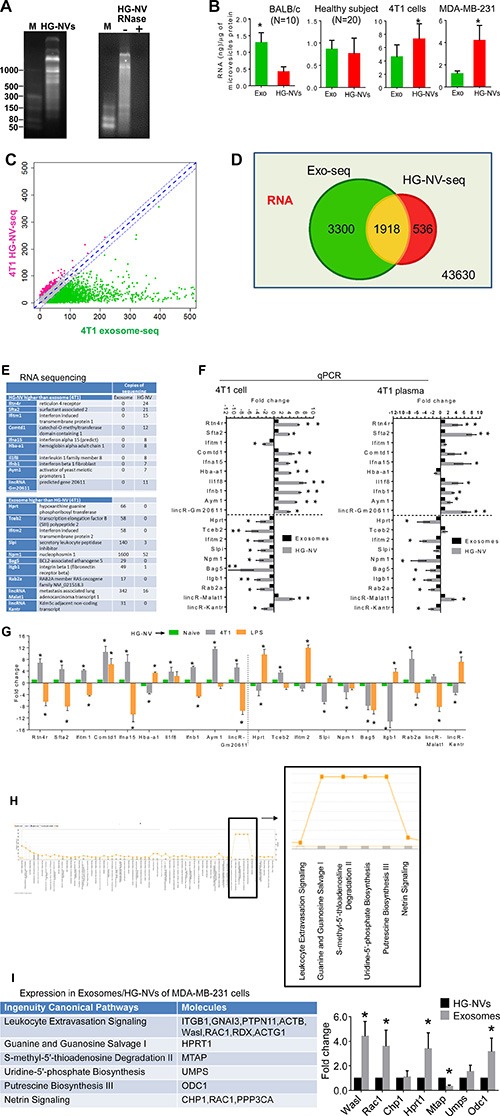
Characterization of tumor cell derived HG-NV RNA (**A**) After electrophoresis on a 12% polyacrylamide gel, HG-NV RNA pretreated with/without RNase was stained with ethidium bromide and visualized using a UVP PhotoDoc-It^™^ Imaging System. (**B**) Total RNA from HG-NVs and exosomes was quantified using Nanodrop spectrophotometry to measure absorbance at 260 nm, and expressed as ng/μg of microvesicle protein (B) Error bars represent standard deviation (± SD) (**p < 0.01). “N” represents the number of samples analyzed. (**C**) XY-scatter plot shows the log2 transformed read counts of RNA sequencing data (Supplementary Table 2) between exosomes (X-axis) and HG-NVs (Y-axis) purified from 4T1 cells. The red dots represent RNAs that are higher in HG-NVs than in exosomes (Differential expression of log2 value > 2), the green dots represent the RNAs that are higher in exosomes than in HG-NVs, the blue dots represent similar levels of RNAs detected in exosomes and HG-NVs. Venn diagram (**D**) shows comparative RNA overlap of the HG-NVs and exosomes. (**E**) Listed RNAs randomly selected from the Supplementary Table 2 are predominately presented in the HG-NVs (upper panel) or vice versa (bottom panel). (**F**) Real-time PCR quantitation of RNAs isolated from HG-NVs and exosomes of 4T1 cell line (left panel) or plasma of 3-week 4T tumor bearing mice (right panel). Fold changes of HG-NV RNA were expressed as the levels of HG-NV RNA/exosomes RNA. *P < 0.05 and **P < 0.01 (two-tailed *t*-test). Data are representative of three independent experiments (*n* = 3 error bars, SEM.). (**G**) Real-time PCR quantitation of RNAs isolated from peripheral blood HG-NVs of naïve mice, 21 day 4T1 tumor bearing mice, and 24 h LPS challenged mice. Fold changes of HG-NV RNA were expressed as the levels of HG-NV RNA from 4T1 tumor bearing mice or LPS challenged mice/PBS treated mice (naïve mice). *P < 0.05 and **P < 0.01 (two-tailed *t*-test). Data are representative of two independent experiments (*n* = 5 error bars, SEM). (**H**) Approximately 300 RNAs that are 5-fold or above lower in HG-HVs than in exosomes were selected and analyzed with ingenuity pathway analysis (IPA). The pathways that are regulated by HG-NV derived RNAs are boxed. (**I**) Listed RNAs isolated from MDA-MB-231HG-NV and exosomes were quantified using real-time PCR. *P < 0.05 (two-tailed *t*-test). Data are representative of three independent experiments (*n* = 3 error bars, SEM.).

Most cells release extracellular vesicles (EVs) containing RNAs, proteins, and lipids [3, 23–25]. To determine whether HG-NVs contain RNA, we took the HG-NVs and exosomes from 4T1 cells and isolated their RNA. Substantial amounts of small-sized RNAs were detected by gel electrophoresis. The HG-NV RNA was found to be resistant to RNase treatment (Figure 2A, right panel). Next, the amounts of RNAs from HG-NVs were compared with the amounts of RNAs in exosomes. Interestingly, although the amounts of HG-NV RNAs from naïve mouse plasma is less than those from exosomes, there is no difference in the levels of RNA present in the HG-NVs and exosomes from the plasma of healthy subjects (Figure 2B). However, the amounts of RNA extracted from HG-NVs of 4T1 cells and the MDA-MB-231 human breast tumor cells were higher than the amounts of RNAs extracted from their exosomes (Figure 2B, right two panels).

To examine if the RNAs were unique to or common between exosomes and HG-NVs, we sequenced RNA from 4T1 HG-NVs and exosomes (Supplementary Table 2). For RNA data analysis, we first removed the low abundant RNAs (< 4 normalized counts per million RNA reads) and then compared the remaining RNAs between 4T1 exosomes and HG-NVs (Figure 2C). Of these, 1,918 were detected in both exosomes and HG-NVs (Figure 2D). In addition to the RNAs that were shared, we also identified some RNAs that were unique to HG-NVs (536) and exosomes (3,300). To validate the RNA sequencing data, we performed a qPCR analysis of 20 RNAs that were randomly selected from the RNA profile that were present or absent in HG-NVs in comparison to exosomes. The data (18/20 RNAs) (Figure 2E–2F) from qPCR were consistent with the data generated from RNA sequencing. Next we determined whether the PCR data generated from the 4T1 cell line could be repeated in an animal model for potential use as biomarkers for disease diagnosis. HG-NVs and exosomes were isolated from the plasma of 4T1 tumor bearing mice. The data (Figure 2F, right panel, 17/20 RNAs) from qPCR were consistent with the data generated from the 4T1 cell line. Then, we further determined whether the HG-NV RNAs that increased in 4T1 tumor bearing mice was disease specific by a comparing the results with a LPS induced inflammation model. The reason we used a LPS induced inflammation mouse model is because inflammation has been known to be involved in the development and progression of numerous diseases. Fifteen out of 20 HG-NV RNAs are 4T1 tumor specific. Eight of 15 of HG-NV RNAs are increased in the plasma of 4T1 tumor bearing and 7/15 are decreased in comparison with HG-NV RNAs in the plasma of LPS challenged mice. Collectively, the PCR data suggest that these HG-NV RNAs could potentially be used as a biomarker for disease diagnosis. The data generated from ingenuity path analysis (IPA) of 4T1 HG-NVs and exosome RNA profiles suggested that the most abundant functions for HG-NV RNAs (Figure 2H) were altered and related to the biosynthesis pathways of guanine/guanosine, adenosine/uridine and putrescine biosynthesis III. This conclusion is also supported by real-time PCR results generated from MDA-MB-231 exosome/HG-NV RNA. Seven randomly selected RNAs that are involved in the biosynthesis pathways of guanine/guanosine, adenosine/uridine and putrescine biosynthesis III were quantitatively analyzed with real-time PCR. The results indicate that 6/7 of HG-NV genes are decreased in the MDA-MB-231 HG-NV in comparison to MDA-MB-231 exosomes (Figure 2I).

### Identification of HG-NV protein composition

**Figure 3:**
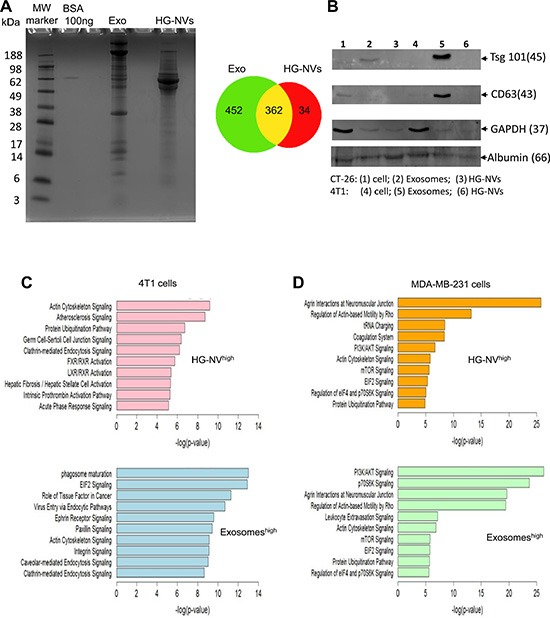
Characterization of tumor cell derived HG-NV proteins (**A**) After electrophoresis on the 8% SDS polyacrylamide gel, the gel was stained with Coomassie Blue and scanned using an Odyssey Imaging System: representative images of the stained gel are shown (left panel), and a Venn diagram (right panel) shows comparative protein overlap of the HG-NVs and exosomes. (**B**) TSG101, CD63, albumin, and GAPDH expression was analyzed by Western blotting. Approximately 200–300 genes that are highly expressed in 4T1 (**C**) or MDA-MB-231 (**D**) HG-NVs or exosomes were analyzed with ingenuity path analysis (IPA). The graph shows the top ten canonical pathways that are regulated by 4T1 or MDA-MB-231HG-NV and exosome derived genes. The x-axis represents -log (*p*-value), where multiple-testing corrected *p*-values were obtained using the Benjamini Hochberg method and represent the significant enrichment of uploaded genes in the functional and canonical pathways shown in Y-axis.

Shown in Figure 3A (left panel) is the migration pattern of 4T1 EV proteins stained with Coomassie blue and Supplementary Table 3 is a listing of the proteins. A total of 848 proteins were identified in the 4T1 EV proteome. In general, many of the proteins identified contained two or more unique peptide hits. Supplementary Table 3 contains detailed information on all of the proteins identified for exosomes and HG-NVs, including the number of unique peptides identified per protein. The pie chart (Figure 3A, right panel) shows that of these proteins, 362 were common to both exosomes and HG-NVs. Furthermore a total of 452 unique proteins were identified in exosomes and 34 unique proteins were identified in HG-NVs (Figure 3A, right panel). To validate the protein data generated from MS/MS analysis, we performed a western blot analysis of proteins that were randomly selected from the protein profile that were increased or decreased in HG-NVs in comparison with exosomes. Western blot analysis (Figure 3B) indicated that both TSG101 and CD63, both of which are considered as exosomal markers, were enriched in exosomes. Albumin was detected in both the exosomes and HG-NVs, suggesting that an equal amount of protein was loaded which validates the western blot results. A higher level of GAPDH was detected in the cell lysates, suggesting that exosomes CD63 and Tsg101 are selectively sorted into the exosomes. We attempted but have not confirmed at this time the specific proteins identified in HG-NVs by MS/MS analysis, which could be due to not having the commercial antibodies available or the lack of specificity for the antibodies available.

Using the IPA software, we classified the proteins that are enriched in HG-NVs or exosomes based on biological function. The top functions for HG-NV proteins (Figure 3C, upper panel) are related to atherosclerosis signaling, ubiquitination and FXR/LXR/RXR mediated signaling pathways. The top functions for exosomal proteins are related to phagosome maturation and EIF2 signaling pathways (Figure 3C, bottom panel). The clathrin-mediated endocytosis signaling pathway is common to both exosomes and HG-NVs. We also classified the proteins which are enriched in MDA-MB-231 HG-NVs or exosomes based on biological function. Supplementary Table 3 contains detailed information on all of the proteins identified for MDA-MB-231 exosomes and HG-NVs, including the number of unique peptides identified per protein. The top functions for MDA-MB-231 HG-NV proteins (Figure 3D, upper panel) are related to tRNA charging and the coagulation system; whereas the predominate function of MDA-MB-231 exosomes was linked to the PI3K and p70S6K mediated signaling pathways (Figure 3D, bottom panel). Agrin interaction at neuromuscular junctions and actin-based mobility signaling pathways are common to both exosomes and HG-NVs.

### ESI-MS/MS profiling and quantitation of 4T1 EV lipids

**Figure 4:**
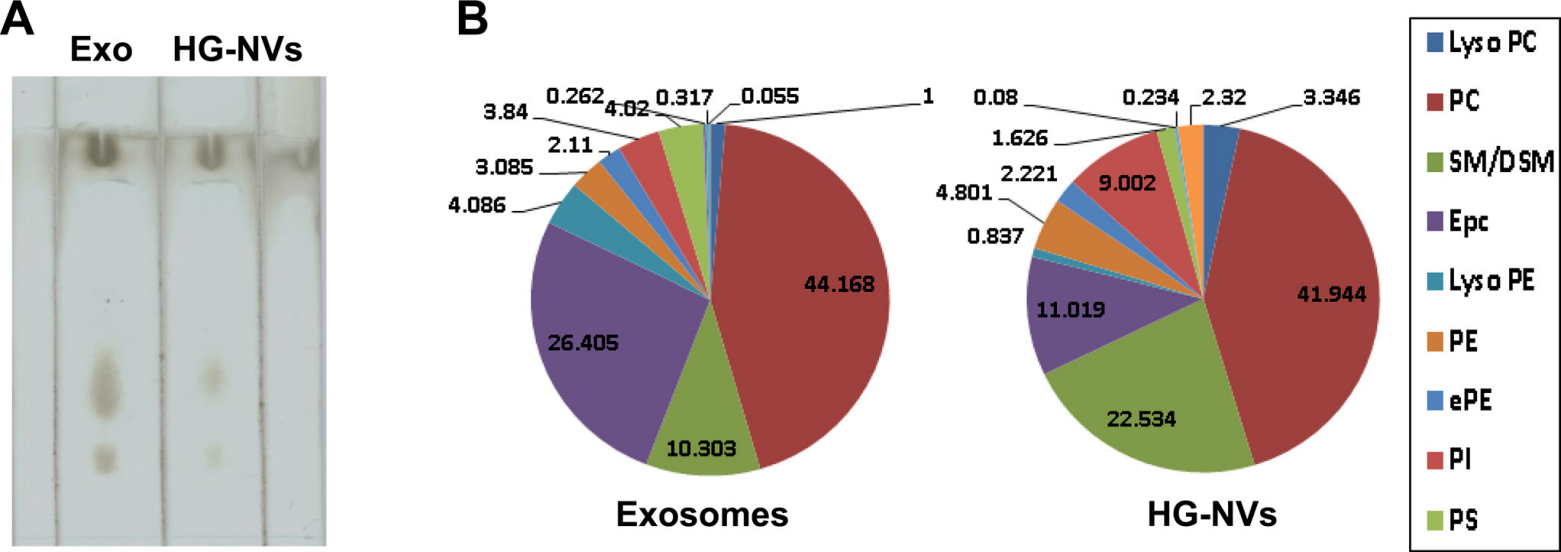
Characterization of tumor cell derived HG-NV lipids Lipids were detected by TLC analysis of the lipid extracts from 4T1 exosomes and HG-NV. The lipids extracted from 4T1 exosomes and HG-NV were separated on a thin-layer chromatography plate and visualized by spraying the plate with a 10% copper sulfate and 8% phosphoric acid solution. (**A**) A representative image was scanned using an Odyssey Scanner. Results represent one of four independent experiments. (**B**) Pie chart with a summary of the putative lipid species in 4T1 exosomes and HG-NVs, reported as percent of total EVs lipids. Major details are reported in Supplementary Table 4 in the Supporting Information. PS: Phosphatidylserine; PI: Phosphatidylinositol; PE: Phosphatidylethanolamines; PC: Phosphatidylcholines; SM/DSM: Mono/Di/ N-(dodecanoyl)-sphing-4-enine-1-phosphocholin.

Electrospray ionization of crude lipid extracts (Figure 4A) from 4T1 exosomes and HG-NVs resulted in the generation of single charged molecular ions with excellent concentration sensitivity. The molecular species of phospholipids present, i.e., PC, PE, PG, PI, PS, PA, lysoPC, and lysoPE, were identified (Supplementary Table 4). The proportion of SM/DSM was twice as high in HG-NVs as in the exosomes; whereas, ePC was much lower in HG-NVs than in exosomes (Figure 4B). An increase of PC and lysoPC and a decrease of lysoPE was observed in HG-NVs in comparison to exosomes (Figure 4B).

### Biological effect of HG-NVs on tumor progression

**Figure 5:**
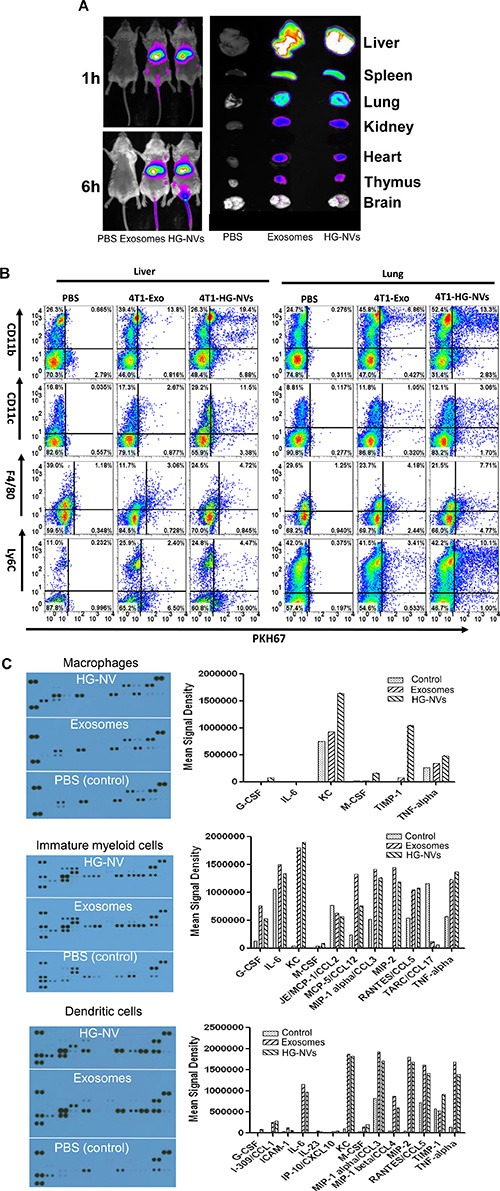
*In vivo* biodistribution of the HG-NV (**A**) Imaging of DiR dye labeled 4T1 exosomes and HG-NV administered intravenously (i.v.) to mice (Left panel) and *in vivo* distribution of DiR dye labeled 4T1 exosomes and HG-NVs determined by scanning (Odyssey scanner) each organ of mice i.v. injected with DiR dye labeled 4T1 exosomes and HG-NVs (right panel). A representative image from each group of mice is shown. (**B**) At 16 h after PKH67 florescent dye labeled 4T1 HG-NVs were administrated intravenously, the percentages of lung and liver leukocytes were quantitatively analyzed by FACS. A representative image is shown. Data (A, B) are representative of at least three independent experiments. (**C**) Inflammatory cytokine expression in HG-NV and exosome stimulated bone marrow derived macrophages (top panel), immature myeloid cells (middle panel) and dendritic cells (bottom panel) was determined using the Proteome Profiler from R&D systems. Each dot represents a cytokine detected by a capture antibody and printed in duplicate on the membrane. The signal intensity of dots on the developed X-ray film was quantified using the LI-COR imaging system and analyzed with LI-COR^®^ Image Studio^™^ Lite Software V3.1.

We next investigated the *in vivo* biological effects of HG-NVs. To determine the tissue tropism of HG-NVs in comparison with exosomes, *in vivo* biodistribution of DiR-labeled HG-NVs or DiR-labeled exosomes was evaluated in mice using a Kodak Image Station 4000 MM Pro system. Six h after a tail-vein injection, DiR fluorescent signals were predominantly detected in the liver, lung, and splenic tissues (Figure 5A). FACS analysis of cells of mice 16 h after receiving an i.v. injection of PKH67-labeled HG-NVs, revealed that higher percentages of CD11c^+^ DCs, F4/80^+^ macrophages and Ly6C^+^ monocytes took up HG-NVs than exosomes (Figure 5B, Table 1).

**Table 1:** Percent of cell up taking 4T1 exosomes and HG-NVs

Cell Type	Liver (*n* = 5)			Lung (*n* = 5)		
	PBS	Exosomes	HG-NVs	PBS	Exosomes	HG-NVs
CD11c^+^PKH26^+^ (DC cells)	0.5 ± 0.1	2.4 ± 0.2	11.2 ± 0.3	0.1 ± 0.1	1.1 ± 0.2	3.1 ± 0.2
F4/80^+^PKH26^+^ (macrophages)	1.1 ± 0.1	3.1 ± 0.3	4.6 ± 0.5	1.3 ± 0.1	4.1 ± 0.4	7.6 ± 0.1
Ly6C^+^PKH26^+^ (monocytes)	0.2 ± 0.1	2.4 ± 0.2	4.5 ± 0.3	0.4 ± 0.1	3.3 ± 0.6	10.3 ± 0.1
CD11b^+^PKH26^+^ (Myeloid cells)	0.7 ± 0.1	13.8 ± 0.4	19.3 ± 0.7	0.3 ± 0.1	6.9 ± 0.3	13.2 ± 0.8

Since the cells targeted by HG-NVs are known to be involved in immune modulation by releasing an array of cytokines, we conducted an analysis of cytokines (Supplementary Figure 2) released from bone marrow derived DCs, macrophages, and immature monocytes after they were stimulated with HG-NVs or exosomes or PBS as a control. Inflammatory cytokine array data (Figure 5C) indicated that the cytokines identified are in much higher concentrations in the cell culture supernatants of macrophages stimulated with HG-NVs for 7 h than with exosomes. MCSF, TIMP1 and KC are increased substantially in HG-NV treated macrophages in comparison to exosome treated macrophages. We also noticed, in general, that stronger inflammatory cytokine signals were detected in the cell culture supernatants of cells treated with either HG-NVs or exosomes than from the PBS control.

Among these three cell types mentioned previously, macrophages are the most abundance in the many different types of tumors and metastatic tissues [26–28]. The upregulated cytokines [29, 30] we detected in culture supernatants of macrophages are known to promote tumor progression. Therefore, we further hypothesize that HG-NVs might enhance or increase tumor progression. Like human breast tumor, 4T1 cells provide an established model of stage IV breast cancer because these cells form tumors when transplanted into mammary glands of mice and spontaneously metastasize to lungs and liver. Therefore, we used the 4T1 murine breast tumor model to test this hypothesis.

**Figure 6:**
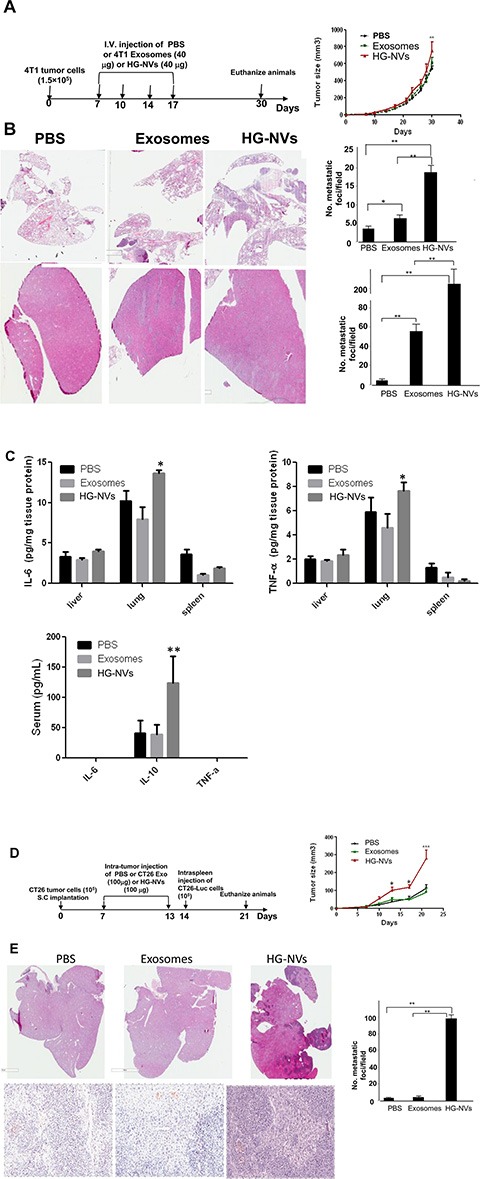
Tumor cell derived HG-NVs promote tumor progression (**A**) Growth curves of 4T1 tumors by orthotopical injection of 4T1 cells into the mammary fat pads in BALB/c mice (5 mice per group) tail-vein injected with 4T1 exosomes or HG-NVs (40 μg/mouse). Schematic representation of injection schedule (a, left panel). Error bars represent standard deviation (± SD) (two-way ANOVA; **p < 0.01). (**B**), A representative photograph showing the H&E stained tissue of 4T1 tumor metastases per field of sectioned lung (Upper panel) and liver (Bottom panel) of 30-day tumor bearing mice. The results are based on three independent experiments (*n* = 5). Means of the number of metastatic foci/field are shown; *P* < 0.001. (**C**), Before mice were sacrificed at day 30 after tumor cells were injected, the levels of IL-6 and TNFα in the lysates from each tissue as labeled in the figure were quantitatively analyzed using an ELISA. The levels of IL-6, IL-10 and TNFα in the sera were also quantitatively analyzed using an ELISA. The results are based on three independent experiments (*n* = 5) and are presented as the mean ± S.E.M.; *p < 0.05, **p < 0.01. (**D**) Growth curves of CT26 tumors after subcutaneously injection of CT26 cells in BALB/c mice (5 mice per group) which were intra-tumorally injected with CT26 exosomes, HG-NV (100 μg/mouse), and PBS as a control. Schematic representation of the injection schedule (d, left panel). Error bars represent standard deviation (± SD) (two-way ANOVA; *p < 0.05, ***p < 0.001). **(E**) A representative photograph shows the H&E stained tissue of CT26 micro-tumors per field of sectioned liver at low magnification (upper panel) and higher magnification (bottom panel) of 21-day tumor bearing mice. The results are based on three independent experiments (*n* = 5).

To investigate whether HG-NVs affected progression of primary and metastatic breast cancer, we injected 1 × 10^4^ 4T1 cells into inguinal mammary fat pads of BALB/c mice. Seven-day tumor bearing mice with similar size tumors were selected and i.v. injected with 4T1 HG-NVs or 4T1 exosomes (40 μg in 50 μl PBS) every three days for 10 days. The host mice displayed visible mammary tumors within two weeks after injection and tumors became necrotic by day 30 which resulted in the experiment being terminated due to Institutional Animal Care and Use Committee guidelines. At day 30 after tumor cells were injected, the tumors in mice receiving HG-NVs increased more rapidly than did tumors in mice receiving exosomes or PBS as a control (Figure 6A). We then sought to determine whether an i.v. injection of 4T1 HG-NVs would promote or increase metastatic occurrence of the tumor. Hematoxylin and eosin staining revealed a significant increase in the number of micro-metastases in the lung (Figure 6B, upper panel) and liver (Figure 6B, bottom panel) compared to exosomes or PBS under the same conditions. ELISA analysis of peripheral blood of mice treated with HG-NVs further revealed a significant increase in TNFα and IL6 detected in the lung and liver tissue lysates and the immunosuppressive cytokine IL-10 (Figure 6C). Collectively, these data indicate that HG-NVs promote early dissemination of the 4T1 cells from primary tumors to lung and liver.

Exosomes released from tumor cells also have a local effect. Published data suggest that exosomes are released into the extracellular tissue space and play a role in tissue remodeling processes [31–33]. Matrix degradation by tumor exosomes has severe consequences on tumor and host cell adhesion, motility, and invasiveness [34]. Our Ki67 FACS analysis results indicate that HG-NVs are more potent in promoting endothelial cell and tumor cell proliferation (Supplementary Figure 3). To address the local effect of HG-NVs on tumor growth, the CT26 colon cancer model was used. The CT26 colon cancer model requires a much longer time for tumor metastasis to occur than the 4T1 model. Therefore, the CT26 colon cancer model is suitable for studying the local effect of HG-NVs in terms of tumor growth before metastasis takes place. Seven-day tumor bearing mice with similar size tumors were treated with CT26 tumor HG-NVs or exosomes or PBS as a control. HG-NVs were injected into the tumor every week for a total of two injections. We then determined their effect on primary colon carcinoma growth. HG-NVs significantly accelerated tumor growth in comparison with exosomes or PBS (Figure 6D), an effect that was evident by day 13 (Figure 6D, right panel *p < 0.05, ***p < 0.001) after the subcutaneous injection of CT-26 tumor cells. On day 14, the tumor volume in the HG-NV treated group was 264.3 ± 38.6 mm^3^, which was significantly larger than tumors in the exosome or PBS treated groups (Figure 6D, ***p < 0.001). We further hypothesized that HG-NV treatment of mice creates a pre-metastatic niche not only by i.v. injection of HG-NVs as shown in Figure 6B but via an intra-tumoral injection as well. To test this hypothesis, one day after the last intra-tumoral injection of HG-NVs, tumor bearing mice were intrasplenic injected with CT26 tumor cells, which is a standard procedure for studying murine colon cancer metastasis to the liver. As shown in Figure 6E, intra-tumor injection of HG-NVs led to a significant increase in the number and size of micro-metastases in the liver compared with exosomes or PBS under the same conditions. However, when NK and T cell deficient NOG mice instead of immunocompetent BALB/c mice were used, no significant differences in terms of tumor growth and liver metastasis was detected (Supplementary Figure 4), indicating that HG-NV-mediated suppression of NK and T cells may be involved in enhancing tumor growth and liver metastasis.

DISCUSSION

In this study, we show that with minimal *in vitro* manipulation only HG-NVs from blood and cell culture supernatants can be detected with a Zetasizer. Five lines of evidence support that HG-NVs are a previously unrecognized nanovesicle. First, unlike exosomes [35, 36], HG-NVs are much smaller in size (8–12 nm in diameter versus 50–150 nm in diameter of exosomes), much less heterogeneous in size and less negatively charged (−10 ± 5 mV) than exosomes (−40 ± 10 mV) released from the same cell types. Second, after depletion of exosomes using a standard protocol, HG-NVs are still present in the samples. Third, based on composition analysis, we identified a number of unique proteins and RNAs being present/absent in the HG-NVs compared with exosomes released from both human and murine breast tumor cells. Fourth, in order to characterize exosomes, they must be concentrated using different technologies that could cause an alteration in their properties [37]. Determining whether the properties of exosomes have actually been altered after *in vitro* concentration is a challenging problem. In contrast, without concentration or other forms of laboratory manipulation, HG-NVs (8–12 nm in diameter) from blood or cell supernatants can be detected with a Nanosizer. Finally, from a biological effects perspective, our data indicate that HG-NVs are different from exosomes (1) in their RNA profile from tumor bearing mice and LPS challenged mice; (2) in their cytokine profile from macrophages, dendritic cells and immature myeloid cells; and (3) in their promoting tumor growth based on two different mouse tumor models used in this study.

Recently, EV-derived molecules have been extensively studied for potential use as biomarkers. In this study, the composition of 4T1 breast tumor cell-derived and MDA-MB-231 human breast tumor cell-derived HG-NVs was further characterized. Besides the proteins and RNAs that are shared among exosomes, the fact that HG-NVs contain much higher copies of specific proteins and RNAs than exosomes released from the same type of tumor cells supports the idea that HG-NV derived RNA and proteins may be used as potential biomarkers for prodiagnosis and diagnosis. This notion was also supported by the specific migration pattern of HG-NV proteins stained with Coomassie dye (Supplementary Figure 5). Furthermore, our data show that one of the biological attributes of the tumor cell-derived HG-NVs is to promote tumor growth and metastasis through immunomodulation. This effect greatly increases the complexity by which tumor cells communicate with immune cells, including macrophages, dendritic cells, and immature myeloid cells that take up HG-NVs as we demonstrated in this study. Cytokines released from macrophages, dendritic cells, and immature myeloid cells participation in immunomodulation in terms of promoting or inhibiting tumor progression and cytokines are major mediators that regulate other immune cell mediated anti-tumor activity including NK, NKT and T cells [38–40]. The results presented in this study indicate that in addition to the identical cytokines induced by exosomes and HG-NVs, some cytokines are only induced by HG-NVs or the exosomes. These cytokines are proinflammatory in nature. A hallmark of tumor progression is the involvement of proinflammatory cytokines [41, 42]. Tumor-associated macrophages [41, 42] and immature myeloid cells [43–45] are the hallmark of immunosuppression in tumors. Therefore, our findings may provide a rationale for developing better cancer immunotherapy strategies by blocking the production of tumor HG-NVs or inhibiting uptake by tumor associated macrophages and immature myeloid cells. Furthermore, given the fact that tumor HG-NV-mediated promotion of lung and liver metastasis did not occur in NOG immune deficient mice, the HG-NVs may have a general role in regulating immune activities of liver, lung and spleen.

In this study, we also demonstrated that one of the characteristics of HG-NVs is that they are much smaller in size than reported for other EVs. In general, the size of a chemically synthesized nanoparticle typically prevents rapid renal clearance (typically must be less than 20 nm) and also prevents uptake by the liver and spleen (typically particles must be greater than 100 nm) [46–51]. However, unlike chemically synthesized nanoparticles, i.v. injected tumor cell-derived HG-NVs do not accumulate in the kidney but do accumulate in lung and liver. Whether the preferential accumulation of HG-NVs in lung and liver is tumor cell specific was not addressed in this study, and requires further study to determine whether HG-NVs from different types of cells under different microenvironments have an effect on *in vivo* distribution and the biological effects on recipient cells.

The finding that HG-NVs are a predominant population among EVs raises a number of important questions to be addressed in the EV field. To date there are almost no data in this field that address the question of whether there is a predominant EV among EVs. Our findings reported in this study provide the basis for further exploring whether HG-NVs are originally released from the same or different compartment of the mother cells as exosomes or whether HG-NVs are originally released from exosomes.

Both exosomes and other EVs could be taken up by the same recipient cells. Currently available isolation and purification methods do not allow one to fully distinguish the biological effect between subpopulations of EVs, and lacking such technology hampers the identification of the *in vivo* physiological relevance and function for each subpopulation. This study demonstrated that tumor cell HG-NVs can be separated from other EVs by differential centrifugation and purified by a simple column based filtration platform. This strategy not only provides a means for investigating the biological effects of HG-NVs released from non-tumor cells under physiological and pathophysiological conditions in general, but could it also provide a possible means to investigate a specific cell type where HG-NVs are detected.

Despite our data that supports that idea that HG-NVs are a predominant subpopulation of EVs, it is important to acknowledge a number of caveats. There is no direct evidence that HG-NVs in the plasma are not contaminated with HDL particles since both are similar in size [52]. However, searching protein profiles from HG-NVs released from mouse and human breast tumor cells, no apolipoprotein A-I (apoA-I), the main protein component of HDL [53], was detected in the HG-NV preparations, suggesting that it is unlikely that the HG-NVs are HDL. Also, it is important to bear in mind that just because a tumor cell-derived HG-NV has the potential to promote tumor progression, that does not mean that other EVs have no role in promoting tumor progression as reported by other groups [19, 21, 24, 54–60]. Instead, this finding opens up a new avenue for further studying whether other types of EVs, which are minor in terms of quantity, have a regulatory role and act in either a synergetic or antagonistic manner with HG-NVs.

## MATERIALS AND METHODS

### Isolation of HG-NV

To characterize the HG-NV, it was necessary to eliminate other subset populations of EVs from the samples. To do this, we saved the supernatants after exosomes had been isolated using a protocol described in the Materials and Methods section, noted as “Exosome isolation”. The isolation and concentration of HG-NVs (HG-nanovesicle isolation system) consists of an Ultrafiltration Biomax-500 (Millipore) and a Masterflex pump with a speed controller. The schematic of the HG-NV isolation system is depicted in Supplementary Figure 1. The supernatants with exosomes depleted were passed through a 0.2 μm filter before loading on the HG-NV isolation system. The supernatants were passed through the Ultrafiltration Biomax-500 column at a flow rate of approximately 3 ml/min, and any molecules less than 500 kDa that passed through the column were collected in a waste jar. Molecules larger than 500 kDa were retained, concentrated, and subjected to sucrose gradient centrifugation.

### Purification of HG-NVs using sucrose gradients

After passing through the HG-NV isolation system, molecules larger than 500 kDa were centrifuged on a 8–45% sucrose density gradient as described previously [61–63]. The purified HG-nanovesicles and exosomes were prepared for EM using a conventional procedure and observed using an FEI Tecnai F20 electron microscope operated at 80 kV and a magnification of 30,000. Photomicrographs were taken using an AMT camera system. Details of other methods used in this study are described in the Supplementary Experimental Procedures.

## SUPPLEMENTARY FIGURES AND TABLES








