# Supplementary material for: Isolation, identification, and characterization of novel nanovesicles
Source: Oncotarget. 2016 May 12;7(27):41346–62. doi: 10.18632/oncotarget.9325 (PMC5173064; doi:10.18632/oncotarget.9325)
Supplement: Supplementary file 1 [file oncotarget-07-41346-s001.pdf]

Isolation, identification, and characterization of novel nanovesicles

Supplementary Materials

MATERIALS AND METHODS

Cell culture

The 4T1 mouse mammary tumor, MDA-MB-231 human breast tumor, CT26 colon tumor and 2H11 endothelial cell lines were purchased from ATCC. Cells were cultured in high glucose DMEM supplemented 10% FBS and antibiotics (100 units/ml penicillin and 100 µg/ml streptomycin) at 37°C in a humidified atmosphere containing 5% CO_2. For exosome and HG-NV isolation, cells were cultured in DMEM supplemented with 10% FBS, previously centrifuged at 100,000 × g overnight to eliminate bovine-derived exosomes. After 24 h in culture, the cells were washed with PBS 2× and cultured for additional 24 h with sera free DMEM medium. The supernatants were harvested for isolating exosomes and HG-NVs and measuring HG-NVs size.

Measurement of particles size and zeta potential

Measurement of the average particle size and particle size distribution, and zeta potential was performed using a Zetasizer Nano ZS (Malvern Instruments, Malvern, UK). The supernatants collected from the cultured cells or plasma collected from mice or human subjects were diluted in PBS before particle size was measured with the Zetasizer Nano ZS according to the instruction described in the manual. Hydrodynamic diameter was determined by dynamic light scattering. The average and standard deviation (SD) were calculated from at least 10 samples. Human samples from healthy subjects and patients were collected in University of Louisville Hospital. Use of human samples in this study was approved by the Institutional Review Board of the University of Louisville Hospital and was conducted in accordance with international guidelines for the use of human tissues.

Exosome isolation

Exosomes were isolated according to a protocol that was described previously [1]. In brief, cell culture medium was subjected to consecutive centrifugation steps (500 g, 2,000 g, 3,000 g and 10,000 g) to remove cellular debris and large vesicles. Exosomes were then pelleted with ultra-centrifugation at 100,000 g (2 hr) and resuspended in PBS for sucrose gradient centrifugation using a method as described [1]. The exosome-depleted supernatants were saved for isolation of HG-NVs. The protein content of the exosomes and HG-NVs were determined using a BCA protein assay kit (Pierce, Rockford, IL, USA).

SDS-PAGE and Western blot analyses

100 µl of each sample (40 µg) were added to an equal volume of boiling 2 × sample buffer and kept at 100°C for 7 min as described previously [1]. The samples were then subjected to 10% SDS-polyacrylamide gel electrophoresis and separated proteins were transferred to nitrocellulose membranes. The western blot was carried out with the anti-CD63, Tsg101, albumin antibodies (Santa Cruz Biotechnology, Santa Cruz, CA) or anti-GAPDH antibody as a control.

RNA extraction and real-time PCR

Total RNA from 4T1 exosomes and HG-NVs was extracted by TRIzol Reagent. Briefly, exosomes and smaller particle samples were homogenized in 1 ml Trizol and incubated at 22°C for 10 min. 0.2 ml of chloroform was mixed and incubated with Trizol reagent at 22°C for 2–3 min. Sample were centrifuged at 12,000 × g for 15 min at 4°C. The aqueous phase was transferred into a new tube, 0.5 ml of 100% isopropanol added to the aqueous phase and the sample incubated at 22°C for 10 min. The sample was centrifuged at 12,000 × g for 10 min at 4°C and the pellet washed with 1 ml of 75% ethanol and dissolved in DEPC treated water for RNA sequencing and real-time PCR analysis. For quantification of genes of interest, RNA (300 ng) was reverse-transcribed with Superscript III and random primers (Invitrogen). cDNA samples were amplified in a CFX96 Realtime System (Bio-Rad Laboratories, Hercules, CA, USA) and Sso Fasteva green supermixture (Bio-Rad Laboratories) according to the manufacturer's instructions. Fold changes in mRNA expression between treatments and controls were determined by the δCT method as described [2]. Differences between groups were determined using a two-sided Student's *t*-test and one-way ANOVA. Error bars on plots represent ± SE, unless otherwise noted. The data were normalized to a GAPDH reference. All primers were purchased from Eurofins MWG Operon. The primer pairs for analysis are provided in Supplementary Table 5. All assays were performed in triplicate a minimum of three times.

To confirm that the nucleic acid isolated from HG-NVs was RNA, nucleic acid from HG-NVs was treated with 1.0 µg/µl RNase (Sigma) or DEPC treated water as a control for 15 min at 37°C before the samples were loaded on a 12% polyacrylamide gel. A total of 1 µg RNA isolated from HG-NVs was resolved on 12% polyacrylamide (*acrylamide/bis-acrylamide*, 29:1) gels containing 8 M Urea and 1 × Tris-Boric Acid-EDTA (TBE, 89 mM tris (pH 7.6), 89 mM boric acid, 2 mM EDTA). After electrophoresis, the gel was stained with ethidium bromide (0.5 µg/ml) and visualized using a UVP PhotoDoc-It™ Imaging System (UVP, Montpelier, Maryland).

RNA sequencing analysis

Total RNA was extracted from 4T1 exosomes and HG-NVs and submitted to the Translational Genomics Research Institute (Phoenix, AZ, USA) for whole transcriptome (WT) sequencing analysis. Briefly, the RNA concentration was measured using Quant-it Ribogreen Assay (Life technologies). 10 ng of RNA were used in the SMARTer Universal Low Input RNA kit (Clontech) for cDNA synthesis. Once cDNA was made, the double stranded cDNA in the samples was measured using Picogreen (Life Technologies). 10 ng ds cDNA were used in the Kapa Biosciences kit, each sample was assigned a unique PCR barcode and 6 PCR cycles were performed for each sample. Samples were then pooled and loaded onto an Illumina HiSeq 2500. For both the WT and small-RNA sequencing, the raw sequence image files from the Illumina HiSeq 2500 in the form of .bcl are converted to the fastq format and checked to ensure the quality scores did not deteriorate drastically at the read ends. The fastqs were trimmed to remove the adapters using Trimmomatic, where the leading and trailing low quality bases below 3 were removed and any reads under the minimum length of 36 nts were discarded. The fastqs were then aligned to the 8059 expressed sequence tags (ESTs) for the 38116 ESTs for Zingiber Officinale from NCBI or the mouse genome (MouseGRCm38, ENSEMBL 79) using STAR v2.4.0j. The STAR aligned sam files are converted to .bam files and sorted by coordinated positions using SAM tools v0.1.19. The read counts were generated using htseq-count (intersection non-empty mode) and the counts for each of the ESTs were generated using idxstats by SAMtools v0.1.19. To validate the RNA sequencing data, we performed a qPCR analysis. The sequences of the primers are listed in Supplementary Table 5. The genes that are significantly higher in HG-NVs than in exosomes were further analyzed using the Ingenuity Pathways Analysis (IPA) software (Ingenuity Systems, Redwood City, CA; http://www.ingenuity.com). Briefly, we uploaded a file containing gene identifiers (ID) and specified mouse as the species. Enrichment of the focus genes (about 300 genes) in the networks were assessed via Fisher's exact test and used to rank the networks. Furthermore, the software identifies top functions and diseases associated with each network via enrichment scores, highlighting the biological significance of the results.

Proteomic analysis

4T1 and MDA-MD-231 exosomes and HG-NVs were lysed in protein lysis buffer and 100 µg of proteins were electrophoresed on 10% SDS-polyacrylamide gels. Coomassie-stained SDS-polyacrylamide gels were cut into 10 strips to correlate with the gel lanes and trypsinized. The digested peptides were loaded on a 100 nm × 10 cm capillary column packed in-house with C18 Monitor 100 A-spherical silica beads and eluted by a 1 h gradient of 10–100% acetonitrile, 0.1% TFA. Mass spectrometry analysis was performed and analyzed using an LTQ XL spectrometer (Thermo Finnigan) at the UAB Proteomic Core Facility. Protein hits were validated using a method as described [3]. Proteins were evaluated by Ingenuity Pathways Analysis software to identify global functions of the proteins. The biological functions assigned to each network were ranked according to the significance of that biological function to the network. A Fischer's exact test was used to calculate a p value. A detailed description of IPA can be found on the Ingenuity Systems website.

Lipidomic analysis

Lipid samples extracted from either 4T1 exosomes or HG-NVs were submitted to the Lipidomics Research Center, Kansas State University (Manhattan, KS) for analysis using a method as described [4]. In brief, the lipid composition was determined using a triple quadrupole mass spectrometer (an Applied Biosystems Q-TRAP, Applied Biosystems, Foster City, CA). The protocol has been previously described [5, 6]. The data are reported as % of total signal for the molecular species determined after normalization of the signals to internal standards of the same lipid class.

TLC analysis

Lipids from ether 4T1 exosomes or HG-NVs were extracted and quantitatively analyzed using a method as described [4]. LC was performed according to the method of Masukawa et al. [7]. Briefly, HPTLC-plates (silica gel 60 with concentrating zone, 20 cm × 10 cm; Merck) were used for the separation. After aliquots of concentrated lipid samples extracted from either 4T1 exosome or HG-NV lipids were separated on a plate, the plate was developed with chloroform/methanol/acetic acid (190:9:1, by vol). After drying, the plates were sprayed with a 10% copper sulfate and 8% phosphoric acid solution and were then charred by heating at 180°C for 5 min. The plate was imaged with an Odyssey Scanner (LI-COR Bioscience, Lincoln NE).

In vitro differentiation of DC and macrophages from BM precursors

BM-derived DC were generated from primary cultures of femoral marrow from 6- to 8-wk-old female wild-type (WT) BALB/c mice as described previously [8]. In brief, BM cells were flushed from the femurs of 6- to 8-wk-old mice using an RPMI 1640-filled syringe to obtain a single-cell suspension. After erythrocytes were depleted, the cells were washed twice with RPMI 1640 (Invitrogen Life Technologies) containing 1% heat-inactivated FBS, and then resuspended in RPMI 1640 supplemented with 10% exosomes depleted FBS, 1 mM pyruvate (Sigma-Aldrich), 1× nonessential amino acids (Sigma-Aldrich), 2 mM glutamine (Sigma-Aldrich), 50 nM 2-ME (Invitrogen Life Technologies), and 20 ng/ml recombinant mouse GM-CSF and 20 pg/ml mouse IL-4 (PeproTech) for DC differentiation and M-CSF (100 U/ml) for macrophages differentiation. The cells were plated at a density of 2×10^6 cells/ml in 6-well plates and cultured at 37°C in a 5% CO2 atmosphere. After 7 days in *ex vivo* culture, the differentiated DCs (> 90% $CD11b^+CD11c^+$) and macrophages (90% > $CD11b^+F4/80^+$) determined by FACS analysis were treated with 4T1 exosomes or HG-NVs (10 µg/ml). Seven hours after the treatments, cell culture supernatant was harvested for cytokine array assay.

In vitro culture immature myeloid cells from BM precursors

Bone marrow was isolated and cultured after RBC lysis as described previously [8]. RBC-depleted bone marrow cells were cultured in RPMI 1640 medium containing 10% exosomes depleted FBS with the addition of glutamine, 2-ME, sodium pyruvate, nonessential amino acid, antibiotics (Invitrogen), and GM-CSF (20 ng/ml), and cultured at 37°C in a 5% CO_2 atmosphere. 4T1 exosomes or HG-NVs (10 µg/ml) were added to the BM cell culture medium on days 0, and 3. Seven hours after the last addition of 4T1 exosomes or HG-NV (10 µg/ml), cell culture supernatant was harvested for cytokine array assay as described below.

Cytokines detection in the supernatants of cultured cells

Inflammatory cytokines in the cultured supernatants harvested from BM derived DCs, macrophages, and immature monocytes were detected with Proteome Profiler Mouse Cytokine Array kit (Cat. No. ARY006, R&D System, Minneapolis, MN, USA) according to the manufacturer's protocol. Briefly, the supernatant of samples was collected by centrifugation at 10,000 g for 5 min at 4°C and the total protein was quantified using a NanoDrop 8000. After blocking for 1 h, the membranes were incubated with a mixture of reconstituted Cytokine Array Detection Antibody Cocktail and the supernatant overnight at 4°C. After washing 3×, the membranes were incubated with streptavidin-HRP for 30 min at 22°C. After washing 3×, the membranes were incubated with 1 ml of Chemi Regent Mix for 1–2 min at 22°C before exposing to X-ray film for 1–5 min. The signal intensity of each dot was quantified with LI-COR imaging system and analyzed with LI-COR® Image Studio™ Lite Software V3.1 (Li-COR bioscience, Lincoln, NE).

Mice

Six to 12-week-old BALB/c and NOD-*scidIL-2R*γ^{null} (NOG) mice which lack mature T cells, B cells, or functional NK cells, and are deficient in cytokine signaling were obtained from Jackson Laboratories. All animal procedures were approved by the University of Louisville Institutional Animal Care and Use Committee.

Hematoxylin and eosin (H&E) staining

For histopathology analysis, H&E staining was performed on paraffin-embedded liver and lung sections using a method as described [4, 9].

Tumor cell and endothelial cell proliferation assay

4T1 tumor cells and 2H11 endothelial cells cultured at 80% confluency were treated with 4T1 exosomes or HG-NV (30 µg/ml) for 24 h. Then, the cells were detached from the cell culture plate using trypsin digestion and washed with PBS. The cell pellet was incubated with cold 70% ethanol for 2 h at −20°C. The cells were washed twice with staining buffer (PBS with 1% FBS, 0.09% NaN^3), and stained for 30 min with properly diluted anti-Ki-67 antibody (e-biosciences). Stained cells were washed prior to FACS analysis.

In vivo image of i.v. injected HG-NVs

To determine the distribution of HG-NVs in mice, DiR dye labeled 4T1 HG-NVs (50 µg) were prepared and intravenously injected into mice. The mice were imaged over a 6-hour period using a Carestream Molecular Imaging system (Carestream Health, Woodbridge, CT). For controls, mice (five per group) received DIR dye in PBS at the same concentration for DIR dye-labeled HG-NVs. Images were collected using a high-sensitivity CCD camera with an exposure time of 2 minutes for imaging.

To determine the percentages of leukocytes from liver and lung taking up HG-NVs, BALB/c mice ($n = 5$) were tail-vein injected with 100 µg PKH67 (Sigma) fluorescent dye labeled HG-NVs/mouse in 100 µl of PBS. 16 h after the injection, mice were sacrificed and leukocytes from liver and lung were isolated using a method as described [10]. Isolated cells were stained with anti- CD11C (dendritic cells), F4/80 (macrophages), and CD11b (total myeloid cells) or Ly6C (monocytes). Subsets of populations with $PKH67^+$ cells were defined using

antibodies against CD11c, F4/80, CD11b or Ly6C. All data were analyzed using FlowJo FACS software.

Murine breast cancer and colon cancer models

Xenograft tumor growth models were used to demonstrate the biological effects of tumor cell derived HG-NVs on tumor progression. To generate a mouse model of breast cancer, 5×10^4 4T1 tumor cells per mouse were orthotopically injected into the mammary fat pads. Female BALB/c mice were purchased from the Jackson Laboratory (Bar Harbor, ME). When tumors reached approximately 60 mm^3 in volume, the mice were randomly assigned to different treatment groups and i.v. injected with 4T1 HG-NVs or exosomes. Mice were treated every 3 days for a total of 4 times. Growth of the tumors was measured using a method as described [11]. Within two weeks after the last injection of HG-NVs, the tumors became necrotic, at which time the experiment had to be terminated. Liver and lung metastasis was evaluated on formalin fixed, paraffin embedded tissue. Serial sections of 5 µm thickness were stained with H&E for morphological analysis.

To generate a mouse model of colon cancer, 1×10^5 colon tumor cells were injected subcutaneously per mouse. When tumors reached approximately 50 mm^3 in volume, the mice were randomly assigned to different treatment groups and injected intra-tumor with CT26 HG-NVs or exosomes (100 µg in 30 µl of PBS) on day 0 and 6. At day 1 after the last intra-tumor injection of CT26 HG-NVs or exosomes, a small left abdominal flank incision was made and the spleen was exteriorized for the intra-splenic injection of CT26 tumor cells (1×10^5). The prepared cells were injected into the spleen using a 30-gauge needle. To prevent tumor cell leakage and bleeding, a cotton swab was held over the site of injection for 1 min. The injected spleen was returned to the abdomen and the wound was sutured with 6–0 black silk. For both 4T1 breast cancer and CT26 colon cancer models, liver and lung metastasis was evaluated on formalin fixed, paraffin embedded tissue. Serial sections of 5 µm thickness were stained with H&E for morphological analysis. Growth of the tumors was measured using a method as described [11]. Tumors were measured with a caliper and tumor volumes were calculated using the formula: length × $width^2$ and presented as the mean ± SD. The number of metastatic foci was counted under low-power (10 × objective) in at least 5 randomly selected locations of each H&E stained specimen of liver and lung by 3 observers blinded to the treatment protocol. The number of tumor nodules represents the mean, with error bars representing the SEM.

To determine the effect of HG-NVs and exosomes on the induction of the cytokines IL-6 and TNF-α which both play a role in the inflammatory mediated promotion of tumor progression, was measured on lung and liver tissue lysates using ELISA kits (eBioscience). Removed lung and liver tissue was flash-frozen in liquid nitrogen until subjected to lysis. To obtain tissue lysates, approximately 30 to 50 mg of tissues were minced and sonicated in 500 µl of lysis buffer (50 mM Tris–HCl pH 7.5) containing 100 mM sodium fluoride, 30 mM sodium pyrophosphate, 2 mM sodium molybdate, 1 mM sodium ortho vanadate, 1 mM glycerophosphate, and 1× protease inhibitor cocktail on ice. Samples were centrifuged at 13,000 rpm for 20 minutes at 4°C. Clear supernatant was collected and used for ELISA. Protein quantification in the lysate was done using the bicinchoninic acid (BCA) method. Serum were also collected on the day when mice were sacrificed and used in an ELISA to detect the induction of cytokines IL-6, IL-10 and TNF-α.

Quantification of HG-NV and exosome RNA from mice in a LPS-induced septic shock mouse model and 4T1 tumor bearing mice

Nine week-old BALB/c female mice were intraperitoneally injected with LPS (10 mg/kg of body weight) or PBS as control. Anticoagulated blood samples were collected 18 h after the I.P. injection. HG-NV RNA was extracted from exosome depleted plasma and the levels of HG-NV RNA were quantitatively analyzed with real-PCR assay. Anticoagulated blood samples were collected from 4T1 tumor bearing mice for quantitative analysis of levels of HG-NV and exosome RNA. Fold changes of HG-NV RNA were expressed as the levels of HG-NV RNA from 4T1 tumor bearing mice or LPS challenged mice compared to PBS treated mice (naïve mice).

REFERENCES

1. Liu Y, Xiang X, Zhuang X, Zhang S, Liu C, Cheng Z, Michalek S, Grizzle W, Zhang HG. Contribution of MyD88 to the tumor exosome-mediated induction of myeloid derived suppressor cells. Am J Pathol. 2010; 176: 2490–2499.
2. Xiang X, Zhuang X, Ju S, Zhang S, Jiang H, Mu J, Zhang L, Miller D, Grizzle W, Zhang HG. miR-155 promotes macroscopic tumor formation yet inhibits tumor dissemination from mammary fat pads to the lung by preventing EMT. Oncogene. 2011; 30:3440–3453.
3. Xiang X, Poliakov A, Liu C, Liu Y, Deng ZB, Wang J, Cheng Z, Shah SV, Wang GJ, Zhang L, Grizzle WE, Mobley J, Zhang HG. Induction of myeloid-derived suppressor cells by tumor exosomes. Int J Cancer. 2009; 124:2621–2633.
4. Wang Q, Zhuang X, Mu J, Deng ZB, Jiang H, Zhang L, Xiang X, Wang B, Yan J, Miller D, Zhang HG. Delivery of therapeutic agents by nanoparticles made of grapefruit-derived lipids. Nat Commun. 2013; 4:1867.
5. Mu J, Zhuang X, Wang Q, Jiang H, Deng ZB, Wang B, Zhang L, Kakar S, Jun Y, Miller D, Zhang HG. Interspecies communication between plant and mouse gut host cells through edible plant derived exosome-like nanoparticles. Mol Nutr Food Res. 2014; 58:1561–1573.

6. Ju S, Mu J, Dokland T, Zhuang X, Wang Q, Jiang H, Xiang X, Deng ZB, Wang B, Zhang L, Roth M, Welti R, Mobley J, et al. Grape exosome-like nanoparticles induce intestinal stem cells and protect mice from DSS-induced colitis. Mol Ther. 2013; 21:1345–1357.
7. Masukawa Y, Narita H, Sato H, Naoe A, Kondo N, Sugai Y, Oba T, Homma R, Ishikawa J, Takagi Y, Kitahara T. Comprehensive quantification of ceramide species in human stratum corneum. J Lipid Res. 2009; 50:1708–1719.
8. Yu S, Liu C, Su K, Wang J, Liu Y, Zhang L, Li C, Cong Y, Kimberly R, Grizzle WE, Falkson C, Zhang HG. Tumor exosomes inhibit differentiation of bone marrow dendritic cells. J Immunol. 2007; 178:6867–6875.
9. Wang B, Zhuang X, Deng ZB, Jiang H, Mu J, Wang Q, Xiang X, Guo H, Zhang L, Dryden G, Yan J, Miller D, Zhang HG. Targeted drug delivery to intestinal macrophages by bioactive nanovesicles released from grapefruit. Mol Ther. 2014; 22:522–534.
10. Liu C, Yu S, Zinn K, Wang J, Zhang L, Jia Y, Kappes JC, Barnes S, Kimberly RP, Grizzle WE, Zhang HG. Murine mammary carcinoma exosomes promote tumor growth by suppression of NK cell function. J Immunol. 2006; 176:1375–1385.
11. Wang Q, Ren Y, Mu J, Egilmez NK, Zhuang X, Deng Z, Zhang L, Yan J, Miller D, Zhang HG. Grapefruit-Derived Nanovectors Use an Activated Leukocyte Trafficking Pathway to Deliver Therapeutic Agents to Inflammatory Tumor Sites. Cancer Res. 2015; 75:2520–2529.

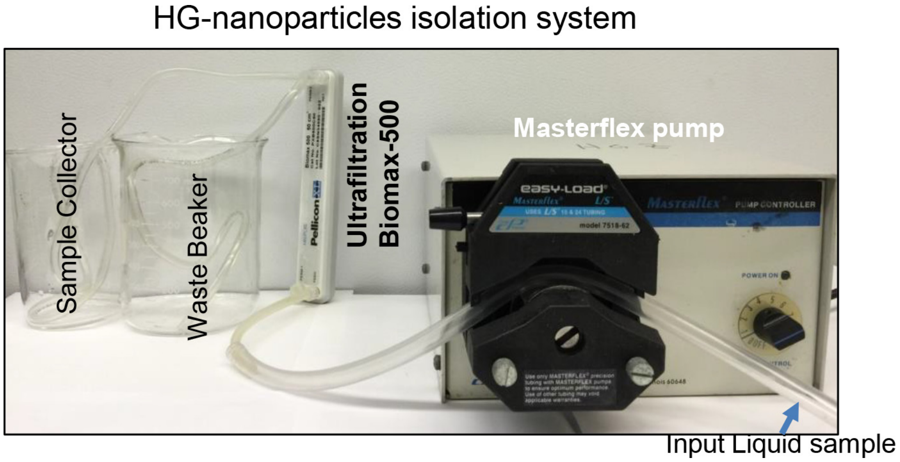

Supplementary Figure S1: The workstation setup for HG-NV isolation. The sample is continually pumped through the plastic tube using a pressure-regulated pump into the Biomax 500 column. The molecules > 500 kDas are retained and collected in the "sample collector", and the molecules < 500 kDas pass through the column and are collected in the "Waste Beaker'.

Location	Analyte	Location	Analyte
A1	Positive Control	C8	IL-12p70
A2	Blank	C9	IL-16
A3	Blank	C10	IL-17
A4	Blank	C11	IL-23
A5	Blank	C12	IL-27
A6	Blank	D1	IP-10/CXCL10
A7	Blank	D2	I-TAC/CXCL11
A8	Blank	D3	KC
A9	Blank	D4	M-CSF
A10	Blank	D5	JE/MCP-1/CCL2
A11	Blank	D6	MCP-5/CCL12
A12	Positive Control	D7	MIG/CXCL9
B1	BLC/BCA-1/CXCL13	D8	MIP-1 alpha/CCL3
B2	C5a	D9	MIP-1 beta/CCL4
B3	G-CSF	D10	MIP-2
B4	GM-CSF	D11	RANTES/CCL5
B5	I-309/CCL1	D12	SDF-1/CXCL12
B6	Eotaxin/CCL11	E1	TARC/CCL17
B7	ICAM-1	E2	TIMP-1
B8	IFN-gamma	E3	TNF-alpha
B9	IL-1 alpha	E4	TREM-1
B10	IL-1 beta	E5	Blank
B11	IL-1ra	E6	Blank
B12	IL-2	E7	Blank
C1	IL-3	E8	Blank
C2	IL-4	E9	Blank
C3	IL-5	E10	Blank
C4	IL-6	E11	Blank
C5	IL-7	E12	Blank
C6	IL-10	F1	Positive Control
C7	IL-13		

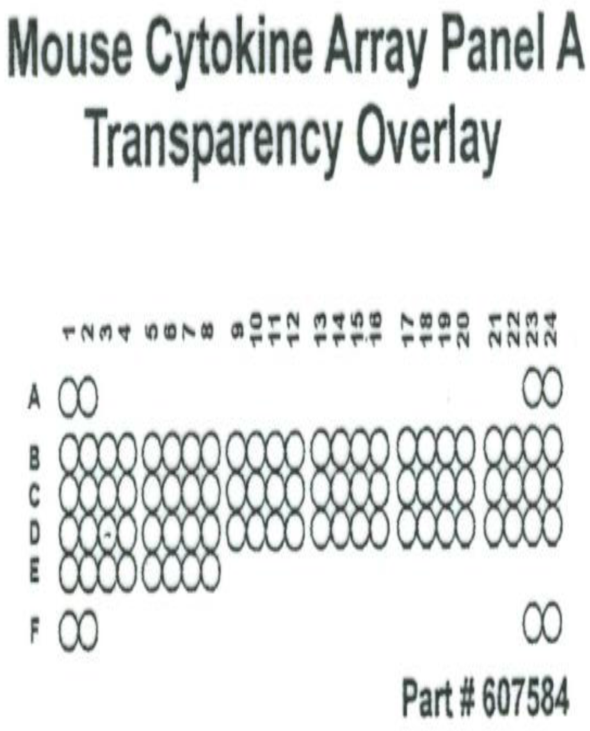

Supplementary Figure S2: Selected capture antibodies as listed (left panel) were spotted in duplicate on nitrocellulose membranes. The positive signals seen on developed film can be quickly identified by placing the transparency overlay template on the array image and aligning it with the pairs of reference spots in three corners of each array.

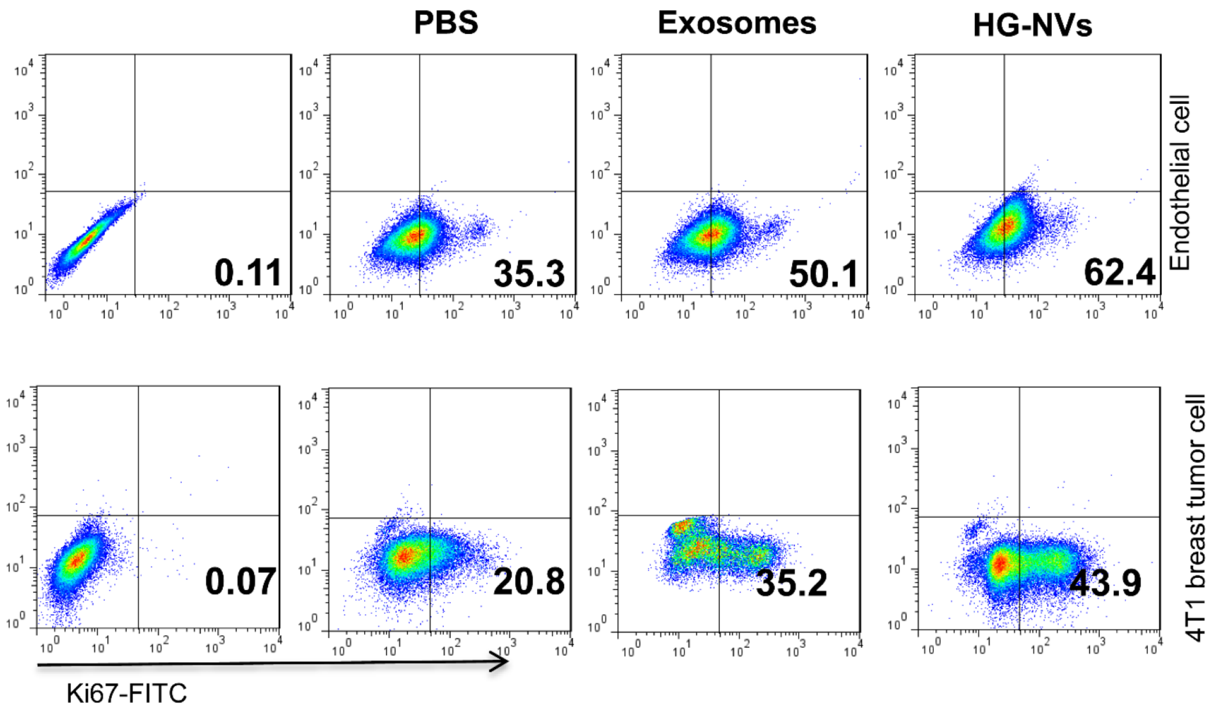

Supplementary Figure S3: 4T1 HG-NVs are more potent in promoting the proliferation of tumor cells and endothelial cells. 24 h after mouse endothelial cells (upper panel) or 4T1 tumor cells were co-cultured with 4T1 HG-NVs and exosomes, the percentages of Ki67+ cells were quantitatively analyzed by FACS. A representative image is shown. Data are representative of three independent experiments (*n* = 5).

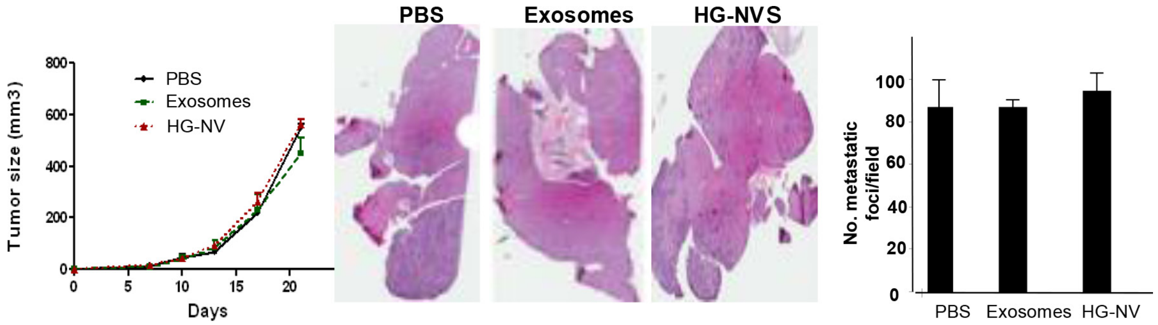

Supplementary Figure S4: Growth curves of CT26 tumors (left panel) by subcutaneously injection of CT26 cells in NK and T cell deficient NOG mice (5 mice per group) which have been intratumoral injected with CT26 exosomes or HG-NVs(100 µg/mouse). The results are presented as the mean ± S.E.M. of independent experiments. A representative photograph shows the H&E stained tissue of CT26 micro-tumors per field of sectioned liver (right panel) of 21 day tumor bearing mice (*n* = 5). Means of the number of liver metastatic foci/field are shown; $P < 0.001$

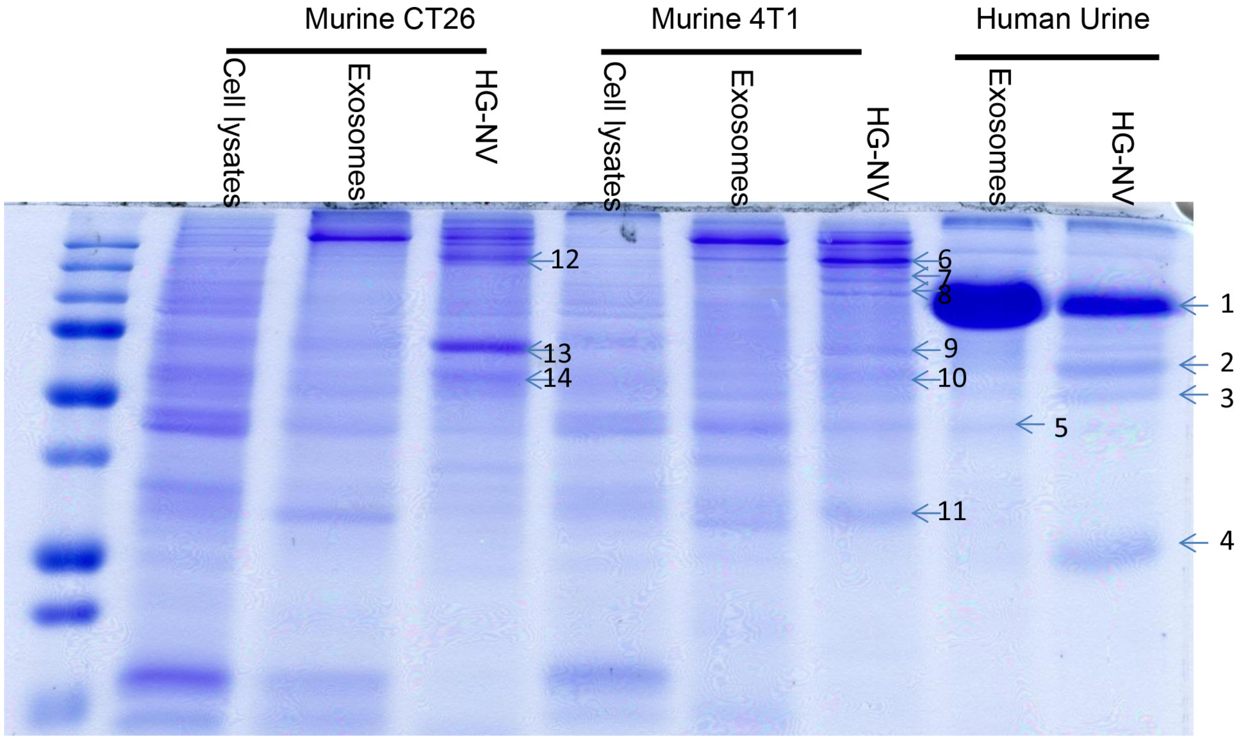

Supplementary Figure S5: After electrophoresis on the 8% SDS polyacrylamide gel, protein lysates (50 µg/lane) extracted from cells, exosomes, HG-NVs were stained with Coomassie Blue and scanned using an Odyssey Imaging System: representative images of the stained gel are shown.

Supplementary Table 1: The size of peripheral blood HG-NV (nm)

		0 h	LPS 6 h	LPS 24 h
C57BL/6	M	7.489 ± 2.238	7.449 ± 1.70	8.212 ± 2.122
	F	7.242 ± 1.465	7.254 ± 1.732	7.518 ± 1.975
	P	8.409 ± 2.298	7.108 ± 1.811	8.193 ± 2.332
BALB/C	M	8.79 ± 2.068	9.481 ± 2.054	6.195 ± 1.622
	F	8.60 ± 2.049	9.18 ± 1.869	7.312 ± 1.918
	P	6.29 ± 1.834	8.375 ± 1.988	7.78 ± 2.042
SCID	M	8.214 ± 2.153	8.754 ± 2.183	7.692 ± 2.225
	F	7.303 ± 1.873	7.336 ± 1.992	6.769 ± 1.931

Note: Plasma collected from mice (n = 5) as listed in the first column were diluted in PBS before the particles' size were measured with Zetasizer Nano ZS. M = male, F = female, and P = pregnancy, SCID = NK and T cell immune deficient NSG mice. Peripheral blood was collected at 0, 6, and 24 h after mice were i.p. injected with LPS (18.5 mg/kg, Sigma-Aldrich).

Supplementary Table S2A: High-level RNA detected in exosome of 4T1. See Supplementary_Table_S2

Supplementary Table S2B: High-level RNA detected in HG-NV of 4T1. See Supplementary_Table_S2

Supplementary Table S3A: High-level protein detected in exosome of 4T1 with proteomics analysis by HPLC-MS. See Supplementary_Table_S3

Supplementary Table S3B: High-level protein detected in HG-NV of 4T1 with proteomics analysis by HPLC-MS. See Supplementary_Table_S3

Supplementary Table 3C: Hige expression in exosomes of MDA-MB-231 cells with proteomics analysis by HPLC-MS. See Supplementary_Table_S3

Supplementary Table S3D: Hige expression in HG-NV of MDA-MB-231 cells with proteomics analysis by HPLC-MS. See Supplementary_Table_S3

Supplementary Table S4: Identities of lipids detected in Exosome/HG-NV. See Supplementary_Table_S4

Supplementary Table S5: Primer sequences used for quantitative Real-Time PCR (qPCR) of mRNA

Gene name	Forward (5′–3′)	Reverse (5′–3′)
Rtn4r	TGCTGGCATGGGTGTTAT	GGCAGCTTGTTGTTACCTTG
Sfta2	AGGACAAGACCTCCCAGAA	TTCAGGCTCTGCAAGTAAGG
Ifitm1	AGCCTTCTCATTCCTTCCTTATT	CCCAGTACAACCACCTCTTG
Comtd1	GGATTCCATGATGACCTGTGA	GTGAAAGTACCCAGATCCAGAG
Ifna15	TTCCTGATGACCCTGCTAGT	GCTCTCTTGTTCCTGAGGTTATG
Hba-a1	GTGGATCCCGTCAACTTCAA	GCTCACAGAGGCAAGGAAT
Il1f8	CTTTGGACCAAGGCAATGATG	CCCTGTAGTTTCTGGGAGATTG
Ifnb1	GATGACGGAGAAGATGCAGAAG	ACCCAGTGCTGGAGAAATTG
Aym1	CAGAGGACACCACTCAGAAATG	GGTGTGTAGACTCACAAGGTTAG
lincRNA Gm20611	CGCTCATCAAGACCTACTTTCTC	CGGAGCAGAAACCCGAATATAG
Hprt	GGGCTTACCTCACTGCTTTC	ACCTGGTTCATCATCGCTAATC
Tceb2	CTTCACTAGCCAGACAGCAC	TCCTGTGGCTTCATCACATC
Ifitm2	GGTCTGGTCCCTGTTCAATAC	ATCTTCCTGTCCCTAGACTTCA
Slpi	AAGTGCGTGAATCCTGTTCC	GCAGACATTGGGAGGGTTAAG
Npm1	GGATGGAACTCCACCCTTTAC	GTCTCCCTACCATTTCCACATC
Bag5	CCAGTGCTTGTGAAACTGAAC	CTGGATCTCCTGAAGCCTACTA
Itgb1	GAGATCAGGAGAACCACAGAAG	AGGAGAGATCCATAAGGTAGTAGAG
Rab2a	GCAGGAGTCCTTTCGTTCTATC	GTTGAACGTGTCTCTCCTTGT
lincRNA Malat1	CAGGGAAGATGGAAGTGTTAGG	CTGGCCTCGAACTCAGAAAT
lincRNA Kantr	TTAGACCCTACGGAGACTAGAG	GCAGGGTGATGGTTCTAATAATG
Rac1	CGCAGACAGACGTGTTCTTA	CCACGAGGATGATAGGAGTATTG
Wasl	GAGGATGATGATGAGTGGGAAG	ACAGATCAATAGACAGGAAGTAGTG
Chp1	CCGGATCATCAATGCCTTCT	TATCCTCAATGGGTCGGAAATG
Mtap	CGGCGGTGAAGATTGGAATA	GGCTTGCCGAATGGAGTAT
Umps	TTGGCCACTGGAAACTACAC	CTTACTCGGGAGCCAGAAATG
Odc1	CTGTGCAAGCAAGACTGAAATAC	GTCATCATCTGGACTCCGTTAC
