# Supplementary material for: Isolation, identification, and characterization of novel nanovesicles
Source: Oncotarget. 2016 May 12;7(27):41346–62. doi: 10.18632/oncotarget.9325 (PMC5173064; doi:10.18632/oncotarget.9325)
Supplement: Supplementary file 3 [file oncotarget-07-41346-s003.docx]

**Supplementary Table S3A: High-level protein detected in exosome of 4T1 with proteomics analysis by HPLC-MS**

| **Identified Proteins** | **Access #** | **MW (kDa)** | **Counts** | **Identified Proteins** | **Access #** | **MW (kDa)** | **Counts** |
| --- | --- | --- | --- | --- | --- | --- | --- |
| Basement membrane-specific heparan sulfate proteoglycan core protein | E9PZ16 | 470 | 184 | Heat shock protein 9 | Q7TSZ0 | 73 | 11 |
| Anastellin | G5E8M2 | 263 | 171 | Galectin-3-binding protein | Q07797 | 64 | 11 |
| Gag-pro-pol polyprotein | Q1KYM2 | 194 | 82 | Fibulin 2, isoform CRA_c | G5E8B3 | 132 | 11 |
| Serum albumin | P02769 | 69 | 62 | Cytoplasmic dynein 1 heavy chain 1 | Q9JHU4 | 532 | 11 |
| Programmed cell death 6-interacting protein | Q9WU78 | 96 | 46 | Collagen alpha-2(IV) chain | P08122 | 167 | 11 |
| Filamin-A | Q8BTM8 | 281 | 39 | Collagen alpha-1(XVIII) chain | P39061 | 182 | 11 |
| Talin-1 | P26039 | 270 | 36 | Brain acid soluble protein 1 | Q91XV3 | 22 | 11 |
| Protein Ahnak | E9Q616 | 604 | 35 | Annexin A4 | P97429 | 36 | 11 |
| Moesin | P26041 | 68 | 35 | Aldehyde dehydrogenase, mitochondrial | P47738 | 57 | 11 |
| Clathrin heavy chain 1 | Q68FD5 | 192 | 34 | Vacuolar protein sorting-associated protein 28 homolog | Q9D1C8 | 25 | 10 |
| Heat shock cognate 71 kDa protein | P63017 | 71 | 32 | T-complex protein 1 subunit beta | P80314 | 57 | 10 |
| Sodium/potassium-transporting ATPase subunit alpha | Q8VDN2 | 113 | 27 | Putative helicase MOV-10 | D3YVL0 | 114 | 10 |
| Tubulointerstitial nephritis antigen- like | H3BJ97 | 49 | 24 | Prohibitin | P67778 | 30 | 10 |
| Milk fat globule-EGF factor 8 protein, isoform CRA_a | Q3TDU5 | 47 | 24 | Neutral amino acid transporter B(0) | E9PUM8 | 58 | 10 |
| Myosin-9 | Q8VDD5 | 226 | 23 | Myoferlin | Q69ZN7 | 233 | 10 |
| Unconventional myosin-Ic | Q9WTI7 | 122 | 22 | Long-chain-fatty-acid--CoA ligase 4 | Q9QUJ7 | 79 | 10 |
| Melanoma antigen | Q2HJ08 | 74 | 22 | Integrin alpha V | A2AKI5 | 112 | 10 |
| EH domain-containing protein 2 | Q8BH64 | 61 | 22 | Immunoglobulin superfamily member 8 | Q8R366 | 65 | 10 |
| Annexin A5 | P48036 | 36 | 22 | Basigin | P18572 | 42 | 10 |
| Actin, cytoplasmic 1, N-terminally processed | F8WI82 | 42 | 22 | 40S ribosomal protein S3 | P62908 | 27 | 10 |
| Vimentin | P20152 | 54 | 21 | 14-3-3 protein beta/alpha | Q9CQV8 | 28 | 10 |
| Polyadenylate-binding protein 1 | P29341 | 71 | 21 | Vinculin | Q64727 | 117 | 9 |
| Integrin beta-1 | P09055 | 88 | 21 | Vacuolar protein sorting-associated protein 37B | Q8R0J7 | 31 | 9 |
| Annexin A2 | P07356 | 39 | 21 | Serine protease HTRA1 | Q9R118 | 51 | 9 |
| Pyruvate kinase isozymes M1/M2 | P52480 | 58 | 20 | Ras-related protein Ral-B | Q9JIW9 | 23 | 9 |
| Prostaglandin F2 receptor negative regulator | Q9WV91 | 99 | 20 | Prolow-density lipoprotein receptor- related protein | Q91ZX7 | 505 | 9 |
| Annexin | Q3U5N9 | 39 | 20 | Programmed cell death protein 6 | P12815 | 22 | 9 |
| Thrombospondin-1 | P35441 | 130 | 19 | Profilin-1 | P62962 | 15 | 9 |
| Integrin alpha-3 | Q62470 | 117 | 19 | Polyubiquitin-C | P0CG50 | 83 | 9 |
| Heat shock protein HSP 90-beta | P11499 | 83 | 19 | Poly(rC)-binding protein 2 | Q61990 | 38 | 9 |
| EH domain-containing protein 1 | Q9WVK4 | 61 | 19 | Peroxiredoxin-1 | P35700 | 22 | 9 |
| Ras GTPase-activating-like protein IQGAP1 | F6ZJB0 | 191 | 18 | Keratin, type I cytoskeletal 14 | Q61781 | 53 | 9 |
| Elongation factor 1-alpha 1 | P10126 | 50 | 18 | Integrin alpha 6 | Q8CC06 | 120 | 9 |
| Collagen alpha-1(XII) chain | E9PX70 | 334 | 18 | Histone H2B type 1-F/J/L | P10853 | 14 | 9 |
| 78 kDa glucose-regulated protein | P20029 | 72 | 18 | Ezrin | P26040 | 69 | 9 |
| 14-3-3 protein zeta/delta | P63101 | 28 | 18 | EH domain-containing protein 4 | Q9EQP2 | 61 | 9 |
| Guanine nucleotide-binding protein G(i) subunit alpha-2 | P08752 | 40 | 17 | Tyrosine-protein kinase Lyn | P25911 | 59 | 8 |
| Elongation factor 2 | P58252 | 95 | 17 | Transgelin-2 | Q9WVA4 | 22 | 8 |

| ATP synthase subunit alpha, mitochondrial | Q03265 | 60 | 17 | Ras-related protein Rab-11B | P46638 | 24 | 8 |
| --- | --- | --- | --- | --- | --- | --- | --- |
| Annexin A3 | O35639 | 36 | 17 | RAB14 protein | Q50HX0 | 24 | 8 |
| 4F2 cell-surface antigen heavy chain | P10852 | 58 | 17 | Protein Rab1 | H7BX41 | 22 | 8 |
| ATP synthase subunit beta, mitochondrial | P56480 | 56 | 16 | Protein Gm10119 | D3Z6C3 | 30 | 8 |
| Tubulin alpha-1C chain | P68373 | 50 | 15 | Plexin-B2 | B2RXS4 | 206 | 8 |
| Major vault protein | Q9EQK5 | 96 | 15 | Multivesicular body subunit 12A | Q78HU3 | 29 | 8 |
| Glypican-1 | Q9QZF2 | 61 | 15 | Monocarboxylate transporter 1 | P53986 | 53 | 8 |
| ADP/ATP translocase 1 | P48962 | 33 | 15 | Integrin beta-3 | O54890 | 87 | 8 |
| Glyceraldehyde-3-phosphate dehydrogenase | E9PX42 | 36 | 14 | H-2 class I histocompatibility antigen, D-D alpha chain | P01900 | 41 | 8 |
| Erythrocyte band 7 integral membrane protein | P54116 | 31 | 14 | Guanine nucleotide-binding protein  G(s) subunit alpha isoforms XLas | Q6R0H7 | 122 | 8 |
| Ceruloplasmin, isoform CRA_a | G3X8Q5 | 124 | 14 | Gap junction alpha-1 protein | P23242 | 43 | 8 |
| Annexin A11 | P97384 | 54 | 14 | Fructose-bisphosphate aldolase A | P05064 | 39 | 8 |
| Alpha-enolase | P17182 | 47 | 14 | Flotillin 2 | Q5SS83 | 47 | 8 |
| 60 kDa heat shock protein, mitochondrial | P63038 | 61 | 14 | DnaJ homolog subfamily A member 1 | P63037 | 45 | 8 |
| Voltage-dependent anion-selective channel protein 1 | Q60932 | 32 | 13 | Coagulation factor V | O88783 | 247 | 8 |
| Syntenin-1 | O08992 | 32 | 13 | 60S ribosomal protein L7 | P14148 | 31 | 8 |
| Inactive tyrosine-protein kinase 7 | Q8BKG3 | 118 | 13 | 40S ribosomal protein S4, X isoform | Q545F8 | 28 | 8 |
| Flotillin-1 | O08917 | 48 | 13 | 40S ribosomal protein S2 | P25444 | 31 | 8 |
| Ephrin type-A receptor 2 | Q03145 | 109 | 13 | 14-3-3 protein eta | P68510 | 28 | 8 |
| Endophilin-A2 | Q62419 | 42 | 13 | Vacuolar protein sorting-associated protein 37C | Q8R105 | 38 | 7 |
| Tumor susceptibility gene 101 protein | Q61187 | 44 | 12 | Sorting nexin-9 | Q91VH2 | 67 | 7 |
| Rap1A-retro1 | C5H0E8 | 21 | 12 | Sorting nexin 18 | Q8C788 | 68 | 7 |
| Keratin, type II cytoskeletal 5 | D3Z4Y4 | 60 | 12 | Na/K-transporting ATPase subunit b-3 | P97370 | 32 | 7 |
| Complement C3 | P01027 | 186 | 12 | Ras-related protein Rap-2a | Q80ZJ1 | 21 | 7 |
| Chloride intracellular channel protein 1 | Q9Z1Q5 | 27 | 12 | Ras-related protein Rab-5C | P35278 | 23 | 7 |
| Tubulin beta-6 chain | Q922F4 | 50 | 11 | Protein Gm10260 | F5H8M6 | 18 | 7 |
| Transforming protein RhoA | Q9QUI0 | 22 | 11 | Protein Fat1 | E9PYL7 | 506 | 7 |
| Rab GDP dissociation inhibitor beta | Q61598 | 51 | 11 | Protein Farp1 | F8VPU2 | 119 | 7 |
| Peptidyl-prolyl cis-trans isomerase | Q3UAJ1 | 18 | 11 | Prohibitin-2 | E9Q313 | 20 | 7 |
| Malate dehydrogenase, mitochondrial | P08249 | 36 | 11 | Phosphoglycerate mutase 1 | Q9DBJ1 | 29 | 7 |
| Phosphate carrier protein, mitochondrial | Q8VEM8 | 40 | 7 | Ras-related protein Rab-2A | P53994 | 24 | 6 |
| Nras protein | Q4FJP3 | 22 | 7 | Protein FAM49B | Q921M7 | 37 | 6 |
| Nidogen-1 | P10493 | 137 | 7 | Protein disulfide-isomerase A6 | Q3TML0 | 49 | 6 |
| L-lactate dehydrogenase A chain | P06151 | 36 | 7 | Protein disulfide-isomerase A3 | P27773 | 57 | 6 |
| Histone H2A type 1-F | Q8CGP5 | 14 | 7 | Phospholipid scramblase 3 | Q9JIZ9 | 32 | 6 |
| Guanine nucleotide binding protein, alpha 11 | Q91X95 | 42 | 7 | Phosphoglycerate kinase 1 | P09411 | 45 | 6 |
| Glia-derived nexin | Q07235 | 44 | 7 | Peroxidasin homolog | Q3UQ28 | 165 | 6 |
| Galectin-1 | P16045 | 15 | 7 | Nucleoside diphosphate kinase B | Q01768 | 17 | 6 |
| Endoplasmin | P08113 | 92 | 7 | Niban-like protein 1 | Q8R1F1 | 85 | 6 |
| E3 ubiquitin-protein ligase Itchy | Q8C863 | 99 | 7 | Neutral amino acid transporter A | O35874 | 56 | 6 |

| D-3-phosphoglycerate dehydrogenase | Q61753 | 57 | 7 | Map4k4 protein | B7ZNR9 | 138 | 6 |
| --- | --- | --- | --- | --- | --- | --- | --- |
| Cofilin-1 | P18760 | 19 | 7 | IST1 homolog | Q9CX00 | 39 | 6 |
| Charged multivesicular body protein 4b | Q9D8B3 | 25 | 7 | H-2K(D) antigen | O35641 | 41 | 6 |
| Chaperonin containing TCP-1 theta subunit | Q9WVS5 | 60 | 7 | Glypican-4 | P51655 | 63 | 6 |
| Calnexin | P35564 | 67 | 7 | Glutamate dehydrogenase 1, mitochondrial | P26443 | 61 | 6 |
| Basement membrane-specific heparan sulfate proteoglycan core protein | Q05793 | 398 | 7 | Glucose-6-phosphate isomerase | P06745 | 63 | 6 |
| ATP synthase subunit O, mitochondrial | Q9DB20 | 23 | 7 | Fumarate hydratase, mitochondrial | P97807 | 54 | 6 |
| Aspartate aminotransferase, mitochondrial | P05202 | 47 | 7 | Formin-like protein 2 | A2APV2 | 123 | 6 |
| 60S acidic ribosomal protein P0 | P14869 | 34 | 7 | Electron transfer flavoprotein subunit  alpha, mitochondrial | Q99LC5 | 35 | 6 |
| 60 kDa SS-A/Ro ribonucleoprotein | O08848 | 60 | 7 | EGF-like repeat and discoidin I-like domain-containing protein 3 | O35474 | 54 | 6 |
| 5'-nucleotidase | Q61503 | 64 | 7 | Dolichyl-diphosphooligosaccharide-- protein glycosyltransferase subunit 2 | A2ACG7 | 68 | 6 |
| Ubiquitin-like modifier-activating  enzyme 1 | Q02053 | 118 | 6 | Disks large homolog 1 | D3Z3B8 | 92 | 6 |
| Triosephosphate isomerase | P17751 | 32 | 6 | Dihydropyrimidinase-related protein 2 | O08553 | 62 | 6 |
| Transferrin receptor protein 1 | Q62351 | 86 | 6 | Collagen alpha-1(IV) chain | P02463 | 161 | 6 |
| T-complex protein 1 subunit zeta | P80317 | 58 | 6 | Clusterin | Q06890 | 52 | 6 |
| T-complex protein 1 subunit eta | P80313 | 60 | 6 | Bone morphogenetic protein 1 | P98063 | 112 | 6 |
| T-complex protein 1 subunit alpha | P11983 | 60 | 6 | ATP citrate lyase | Q3V117 | 121 | 6 |
| Synaptosomal-associated protein 23 | O09044 | 23 | 6 | Alpha-actinin-4 | P57780 | 105 | 6 |
| Synaptic vesicle membrane protein VAT-1 homolog | Q62465 | 43 | 6 | ADP-ribosylation factor 2 | Q8BSL7 | 21 | 6 |
| Succinyl-CoA:3-ketoacid-coenzyme  A transferase 1, mitochondrial | Q3UJQ9 | 52 | 6 | Adenosylhomocysteinase | P50247 | 48 | 6 |
| S-methyl-5'-thioadenosine phosphorylase | Q9CQ65 | 31 | 6 | Actin-related protein 3 | Q99JY9 | 47 | 6 |
| RuvB-like 1 | P60122 | 50 | 6 | 60S ribosomal protein L18 | Q642K1 | 22 | 6 |
| Rps16 protein | Q5CZY9 | 19 | 6 | 3-hydroxyacyl-CoA dehydrogenase type-2 | A2AFQ2 | 28 | 6 |
| Ras-related protein Rab-5A | Q9CQD1 | 24 | 6 | 14-3-3 protein theta | P68254 | 28 | 6 |

# Supplementary Table S3B: High-level protein detected in HG-NV of 4T1 with proteomics analysis by HPLC-MS

**Identified Proteins Access # MW (kDa)**

**Counts Identified Proteins Access # MW**

**(kDa)**

**Counts**

| Anastellin | G5E8M2 | 263 | 188 | 60S ribosomal protein L3 | P27659 | 46 | 12 |
| --- | --- | --- | --- | --- | --- | --- | --- |
| Basement membrane-specific heparan sulfate proteoglycan core protein | E9PZ16 | 470 | 168 | Alpha-2-macroglobulin-P | Q6GQT1 | 164 | 12 |
| Serum albumin | P02769 | 69 | 162 | 40S ribosomal protein S9 | D3YWH9 | 16 | 12 |
| Collagen alpha-1(XII) chain | E9PX70 | 334 | 64 | Protein Pzp | D3YW52 | 167 | 12 |
| Inter-alpha-trypsin inhibitor heavy chain H2 | Q61703 | 106 | 60 | Laminin subunit alpha-5 | Q61001 | 404 | 12 |
| Actin, cytoplasmic 1, N-terminally processed | F8WI82 | 42 | 52 | Collagen alpha-1(VI) chain | Q04857 | 108 | 12 |
| Heat shock cognate 71 kDa protein | P63017 | 71 | 44 | Beta-globin | A8DUN2 | 16 | 12 |
| Thrombospondin-1 | P35441 | 130 | 44 | Renin receptor | Q9CYN9 | 39 | 12 |
| Programmed cell death 6-interacting protein | Q9WU78 | 96 | 40 | Prothrombin | P19221 | 70 | 12 |
| Talin-1 | P26039 | 270 | 36 | Fibulin-1 | Q08879 | 78 | 12 |
| Complement C3 | P01027 | 186 | 32 | Bromodomain-  containing protein 9 | Q3UQU0 | 67 | 12 |
| Gag-pro-pol polyprotein | Q1KYM2 | 194 | 28 | Beta-glucuronidase | P12265 | 74 | 12 |
| Prostaglandin F2 receptor negative regulator | Q9WV91 | 99 | 28 | Abnormal spindle-like microcephaly-associated protein homolog | Q8CJ27 | 364 | 12 |
| Heat shock protein HSP 90-beta | P11499 | 83 | 28 | Sister chromatid cohesion protein PDS5 homolog B | F8WHU5 | 165 | 12 |
| Elongation factor 1-alpha 1 | P10126 | 50 | 28 | Inter alpha-trypsin inhibitor, heavy chain 4 | A6X935 | 100 | 12 |
| Ceruloplasmin, isoform CRA_a | G3X8Q5 | 124 | 28 | Sodium/potassium- transporting ATPase subunit alpha-1 | Q8VDN2 | 113 | 8 |
| Prolow-density lipoprotein receptor-related protein 1 | Q91ZX7 | 505 | 24 | Unconventional myosin- Ic | Q9WTI7 | 122 | 8 |
| Histone H2A type 1-F | Q8CGP5 | 14 | 24 | Pyruvate kinase isozymes M1/M2 | P52480 | 58 | 8 |
| Serotransferrin | Q921I1 | 77 | 24 | Integrin alpha-3 | Q62470 | 117 | 8 |
| Insulin-like growth factor 2 receptor | B7ZWC4 | 274 | 24 | 78 kDa glucose-regulated protein | P20029 | 72 | 8 |
| Titin | A2ASS6 | 3906 | 24 | Annexin A3 | O35639 | 36 | 8 |
| Tubulointerstitial nephritis antigen-like | H3BJ97 | 49 | 20 | Glypican-1 | Q9QZF2 | 61 | 8 |
| Melanoma antigen | Q2HJ08 | 74 | 20 | 60 kDa heat shock protein, mitochondrial | P63038 | 61 | 8 |
| Integrin beta-1 | P09055 | 88 | 20 | Tubulin beta-6 chain | Q922F4 | 50 | 8 |
| Tubulin alpha-1C chain | P68373 | 50 | 20 | Fibulin 2, isoform CRA_c | G5E8B3 | 132 | 8 |
| Keratin, type II cytoskeletal 5 | D3Z4Y4 | 60 | 20 | Collagen alpha-2(IV) chain | P08122 | 167 | 8 |
| Vinculin | Q64727 | 117 | 20 | Annexin A4 | P97429 | 36 | 8 |

| Serine protease HTRA1 | Q9R118 | 51 | 20 | T-complex protein 1 subunit beta | P80314 | 57 | 8 |
| --- | --- | --- | --- | --- | --- | --- | --- |
| Keratin, type I cytoskeletal 14 | Q61781 | 53 | 20 | Profilin-1 | P62962 | 15 | 8 |
| Histone H2B type 1-F/J/L | P10853 | 14 | 20 | Ras-related protein Rab- 11B | P46638 | 24 | 8 |
| Clusterin | Q06890 | 52 | 20 | 40S ribosomal protein S2 | P25444 | 31 | 8 |
| Keratin, type I cytoskeletal 10 | E9QLP8 | 49 | 20 | Protein Fat1 | E9PYL7 | 506 | 8 |
| Heat shock protein HSP  90-alpha | P07901 | 85 | 20 | Glia-derived nexin | Q07235 | 44 | 8 |
| Protein Apob | E9Q414 | 509 | 20 | Cofilin-1 | P18760 | 19 | 8 |
| Inter-alpha-trypsin inhibitor heavy chain H3 | E9PVS1 | 78 | 20 | Collagen alpha-1(IV) chain | P02463 | 161 | 8 |
| Apoa1 protein | Q58EV2 | 23 | 20 | Bone morphogenetic protein 1 | P98063 | 112 | 8 |
| 14-3-3 protein zeta/delta | P63101 | 28 | 16 | Cell division control protein 42 homolog | Q3UL78 | 17 | 8 |
| Alpha-enolase | P17182 | 47 | 16 | 40S ribosomal protein SA | P14206 | 33 | 8 |
| Chloride intracellular channel protein 1 | Q9Z1Q5 | 27 | 16 | Tubulin beta-3 chain | Q9ERD7 | 50 | 8 |
| Galectin-3-binding protein | Q07797 | 64 | 16 | Proteasome subunit alpha type-4 | Q9R1P0 | 29 | 8 |
| 60S acidic ribosomal protein P0 | P14869 | 34 | 16 | Proteasome subunit alpha type-2 | P49722 | 26 | 8 |
| Keratin, type II cytoskeletal 1 | P04104 | 66 | 16 | Keratin, type II cytoskeletal 8 | P11679 | 55 | 8 |
| Krt2 protein | B2RTP7 | 71 | 16 | Ferritin | Q3TJJ6 | 21 | 8 |
| Antithrombin-III | P32261 | 52 | 16 | Pigment epithelium- derived factor | P97298 | 46 | 8 |
| Serum albumin | P07724 | 69 | 16 | Peptidyl-prolyl cis-trans isomerase B | P24369 | 24 | 8 |
| Type VI collagen alpha 3 subunit | O88493 | 287 | 16 | Histone H3 | F8WI35 | 15 | 8 |
| Thrombospondin-4 | Q9Z1T2 | 106 | 16 | CD 81 antigen, isoform CRA_c | Q91V78 | 26 | 8 |
| Nucleophosmin | Q9DAY9 | 28 | 16 | Serine/arginine-rich- splicing factor 1 | H7BX95 | 28 | 8 |
| Laminin B1 subunit 1 | B9EKB0 | 202 | 16 | Regucalcin | Q64374 | 33 | 8 |
| Procollagen C-endopeptidase enhancer 1 | Q61398 | 50 | 16 | Protein Krt78 | E9Q0F0 | 112 | 8 |
| Insulin-like growth factor- binding protein 4 | P47879 | 28 | 16 | Protein Hba-a1 | F7CAE1 | 15 | 8 |
| Collagen alpha-1(I) chain | P11087 | 138 | 16 | Proteasome subunit beta type-3 | Q9R1P1 | 23 | 8 |
| Milk fat globule-EGF factor 8 protein, isoform CRA_a | Q3TDU5 | 47 | 12 | Proteasome subunit alpha type-3 | O70435 | 28 | 8 |
| Annexin A2 | P07356 | 39 | 12 | Nascent polypeptide- associated complex subunit a, muscle- specific form | P70670 | 220 | 8 |
| ATP synthase subunit beta, mitochondrial | P56480 | 56 | 12 | Lumican | P51885 | 38 | 8 |

| Glyceraldehyde-3-phosphate dehydrogenase | E9PX42 | 36 | 12 | Histone H2A | Q5NC91 | 9 | 8 |
| --- | --- | --- | --- | --- | --- | --- | --- |
| Rap1A-retro1 | C5H0E8 | 21 | 12 | Gelsolin | P13020 | 86 | 8 |
| Cytoplasmic dynein 1 heavy chain 1 | Q9JHU4 | 532 | 12 | Try10-like trypsinogen | Q7M754 | 27 | 8 |
| Collagen alpha-1(XVIII) chain | P39061 | 182 | 12 | Terminal uridylyltransferase 4 | B2RX14 | 185 | 8 |
| Polyubiquitin-C | P0CG50 | 83 | 12 | Proviral envelope protein | P97406  (+2) | 66 | 8 |
| Coagulation factor V | O88783 | 247 | 12 | Protein Trp53bp1 | A2AU91 | 213 | 8 |
| Basement membrane-specific heparan sulfate proteoglycan core protein | Q05793 | 398 | 12 | Osteopontin | F8WIP8 | 33 | 8 |
| Triosephosphate isomerase | P17751 | 32 | 12 | Ninein-like protein | Q6ZQ12 | 158 | 8 |
| Adenosylhomocysteinase | P50247 | 48 | 12 | Keratin, type II cytoskeletal 73 | Q6NXH9 | 59 | 8 |
| 60S ribosomal protein L18 | Q642K1 | 22 | 12 | Keratin, type II cytoskeletal 72 | Q6IME9 | 57 | 8 |
| Nucleolin | P09405 | 77 | 12 | Keratin, type II cytoskeletal 6A | P50446 | 59 | 8 |
| Keratin, type II cytoskeletal 2 oral | Q3UV17 | 63 | 8 | Pecanex-like protein 1 | E9QPL4 | 248 | 6 |
| Glutaminyl-tRNA synthetase | Q8BML9 | 88 | 8 | Pantetheinase | Q9Z0K8 | 57 | 6 |
| E3 SUMO-protein ligase RanBP2 | Q9ERU9 | 341 | 8 | Olfactory receptor 303 | Q8VFP0 | 36 | 6 |
| Complement component 4B (Childo blood group) | B2RWX2 | 193 | 8 | Nipped-B-like protein | Q6KCD5 | 315 | 6 |
| Complement C1q tumor necrosis factor-related protein 3 | D3YZ61 | 35 | 8 | Neurofilament heavy  polypeptide | P19246 | 117 | 6 |
| Collagen alpha-2(I) chain | Q01149 | 130 | 8 | Methyl-CpG-binding protein 2 | Q9Z2D6 | 52 | 6 |
| Citrate synthase | Q80X68 | 52 | 8 | Lysosomal alpha- mannosidase | O09159 | 115 | 6 |
| Beta-2-microglobulin | P01887 | 14 | 8 | Lysine-specific  demethylase 2B | D3YVU4  (+2) | 146 | 6 |
| U2 snRNP-associated SURP motif-containing protein | Q6NV83 | 118 | 8 | Lipoxygenase homology domain-containing protein 1 | C8YR32 | 236 | 6 |
| Synaptonemal complex protein 1 | Q62209 | 116 | 8 | Lactoferrin | B8YJF9 | 78 | 6 |
| Spatacsin | Q3UHA3 | 274 | 8 | Keratin, type I cytoskeletal 13 | P08730 | 48 | 6 |
| Sortilin-related receptor | O88307 | 247 | 8 | Homeobox protein unc-4 homolog | O08934 | 54 | 6 |
| Ryanodine receptor 3 | A2AGL3 | 551 | 8 | Histone-lysine  N-methyltransferase MLL | P55200 | 430 | 6 |
| Ryanodine receptor 2 | E9Q401 | 565 | 8 | E3 ubiquitin-protein ligase MIB1 | Q80SY4 | 110 | 6 |
| Rho guanine nucleotide exchange factor 4 | E0CX56 | 68 | 8 | Delta-1-pyrroline-5- carboxylate synthase | Q9Z110 | 87 | 6 |

| Regulator of G-protein- signaling 12 | E9Q652 | 157 | 8 | Dedicator of cytokinesis protein 4 | F6SJX1 | 227 | 6 |
| --- | --- | --- | --- | --- | --- | --- | --- |
| Putative rRNA methyltransferase 3 | Q9DBE9 | 96 | 7 | Collagen alpha-2(XI)  chain | Q64739 | 172 | 6 |
| Proteoglycan 4 | E0CZ58 | 135 | 7 | Collagen alpha-1(III) chain | F6SIG2 | 115 | 6 |
| Protein Zfp457 | E9PUC7 | 75 | 7 | Coiled-coil domain- containing protein 38 | Q8CDN8 | 66 | 6 |
| Protein Zfp281 | Q99LI5 | 97 | 7 | Canalicular multispecific organic anion transporter 2 | B2RX12 | 169 | 6 |
| Protein sprouty homolog 1 | Q9QXV9 | 34 | 7 | Cadherin-8 | E9PZC1 | 28 | 6 |
| Protein Neb | E9Q1W3 | 829 | 7 | Brain-specific  angiogenesis inhibitor 3 | Q80ZF8 | 171 | 6 |
| Protein Gm15800 | E9PX61 | 453 | 7 | Biglycan | P28653 | 42 | 6 |
| Protein Fam38a | E9PY63 | 125 | 6 | Anaphase-promoting complex subunit 7 | Q9WVM3 | 63 | 6 |
| Protein FAM205A | A2APU8 | 146 | 6 | AA987161 protein | Q80VN4 | 120 | 6 |

| **Supplementary Table 3c Hige expression in exosomes of MDA-MB-231 cells with proteomics analysis by HPLC-MS** | | | | | |  |  |
| --- | --- | --- | --- | --- | --- | --- | --- |
| **Identified Proteins** | **Access #** | **MW (kDa)** | **Counts** | **Identified Proteins** | **Access #** | **MW (kDa)** | **Counts** |
| Cytoplasmic dynein 1 heavy chain 1 | Q14204 | 532 | 152 | Microtubule-associated protein 1B | P46821 | 271 | 14 |
| Filamin-A | P21333 | 281 | 85 | Eukaryotic translation initiation factor 3 subunit B | P55884 | 92 | 14 |
| Plectin | Q15149 | 532 | 80 | Poly [ADP-ribose] polymerase 1 | P09874 | 113 | 14 |
| Myosin-9 | P35579 | 227 | 75 | Proteasome subunit alpha type-1 | P25786 | 30 | 14 |
| DNA-dependent protein kinase catalytic subunit | P78527 | 469 | 70 | Cell growth inhibiting protein 40 | Q2TTR7 | 134 | 13 |
| Fatty acid synthase | P49327 | 273 | 68 | Ras-related protein Rab-7a | P51149 | 23 | 13 |
| Talin-1 | Q9Y490 | 270 | 60 | Elongation factor 1-delta | P29692 | 31 | 13 |
| Clathrin heavy chain 1 | Q00610 | 192 | 59 | Laminin subunit gamma-1 | P11047 | 178 | 13 |
| Myoferlin | Q9NZM1 | 235 | 53 | 26S protease regulatory subunit 10B | P62333 | 44 | 12 |
| Filamin B | B2ZZ83 | 282 | 47 | DNA topoisomerase 1 | P11387 | 91 | 12 |
| Filamin-C | Q14315 | 291 | 42 | Ephrin type-A receptor 2 | P29317 | 108 | 12 |
| Fibronectin | P02751 | 263 | 38 | Major vault protein | Q14764 | 99 | 12 |
| Bifunctional glutamate/proline--tRNA ligase | P07814 | 171 | 36 | 60 heat shock protein, mitochondrial | P10809 | 61 | 12 |
| Basement membrane-specific heparan sulfate proteoglycan core protein | P98160 | 469 | 34 | 26S protease regulatory subunit 8 | A8K3Z3 | 45 | 12 |
| Translational activator GCN1 | Q92616 | 293 | 33 | Heterogeneous nuclear ribonucleoproteins C1/C2 | P07910 | 34 | 12 |
| Pre-mRNA-processing-splicing factor 8 | Q6P2Q9 | 274 | 32 | RuvB-like 2 | Q9Y230 | 51 | 12 |
| Ras GTPase-activating-like protein IQGAP1 | P46940 | 189 | 30 | 60S ribosomal protein L4 | P36578 | 48 | 11 |
| Heterogeneous nuclear ribonucleoprotein U | Q00839 | 91 | 28 | Cation-independent mannose-6-phosphate receptor | P11717 | 274 | 11 |
| E3 ubiquitin-protein ligase UBR4 | Q5T4S7 | 574 | 27 | 40S ribosomal protein S6 | P62753 | 29 | 11 |
| Chaperonin containing TCP1, subunit 8 (Theta), isoform CRA_a | G5E9B2 | 59 | 27 | 40S ribosomal protein S2 | P15880 | 31 | 11 |
| Programmed cell death 6-interacting protein | Q8WUM4 | 96 | 27 | 26S protease regulatory subunit 4 | P62191 | 49 | 11 |
| Sodium/potassium-transporting ATPase subunit alpha-1 | P05023 | 113 | 27 | Ribosomal protein S27a | Q5RKT7 | 18 | 11 |
| T-complex protein 1 subunit eta | Q99832 | 59 | 27 | Peptidyl-prolyl cis-trans isomerase FKBP4 | Q02790 | 52 | 11 |
| Collagen alpha-1(XII) chain | Q99715 | 333 | 25 | FACT complex subunit SPT16 | Q9Y5B9 | 120 | 11 |
| Isoleucine--tRNA ligase, cytoplasmic | P41252 | 145 | 25 | Spectrin beta chain, brain 1 | Q01082 | 275 | 11 |
| Elongation factor 1-alpha 1 | P68104 | 50 | 25 | 40S ribosomal protein S7 | P62081 | 22 | 10 |
| T-complex protein 1 subunit gamma | P49368 | 61 | 25 | Structural maintenance of chromosomes 3 | Q86VX4 | 142 | 10 |
| EGF-like repeat and discoidin I-like domain-containing protein 3 | O43854 | 54 | 25 | Protein DEK | P35659 | 43 | 10 |
| Annexin | A6NN80 | 75 | 24 | 78 glucose-regulated protein | P11021 | 72 | 10 |
| Leucine--tRNA ligase, cytoplasmic | Q9P2J5 | 134 | 23 | Aminoacyl tRNA synthase complex-interacting multifunctional protein 1 | Q12904 | 34 | 10 |
| U5 small nuclear ribonucleoprotein 200 kDa helicase | O75643 | 245 | 22 | Splicing factor 3B subunit 1 | O75533 | 146 | 10 |
| Proteasome-associated protein ECM29 homolog | Q5VYK3 | 204 | 22 | Glycine--tRNA ligase | P41250 | 83 | 10 |
| Kinesin-1 heavy chain | P33176 | 110 | 21 | HSPC027 | Q9Y6E3 | 43 | 10 |
| E3 ubiquitin-protein ligase HUWE1 | Q7Z6Z7 | 482 | 20 | Multifunctional protein ADE2 | P22234 | 47 | 10 |
| CAD protein | P27708 | 243 | 20 | 14-3-3 protein theta | P27348 | 28 | 10 |
| Tenascin | P24821 | 241 | 20 | Proteasome subunit alpha type-2 | P25787 | 26 | 10 |
| T-complex protein 1 subunit epsilon | P48643 | 60 | 20 | Eukaryotic translation initiation factor 3 subunit D | O15371 | 64 | 10 |
| Elongation factor 1-gamma | P26641 | 50 | 20 | Proteasome subunit beta type-4 | P28070 | 29 | 10 |
| Neuroblast differentiation-associated protein AHNAK | Q09666 | 629 | 19 | Proteasome subunit beta type-5 | P28074 | 28 | 10 |
| T-complex protein 1 subunit zeta | P40227 | 58 | 19 | 60S ribosomal protein L10 | P27635 | 25 | 9 |
| Valine--tRNA ligase | P26640 | 140 | 19 | Mitochondrial import receptor subunit TOM34 | Q15785 | 35 | 9 |
| T-complex protein 1 subunit delta | P50991 | 58 | 18 | Peptidyl-prolyl cis-trans isomerase A | Q567Q0 | 11 | 9 |
| C-1-tetrahydrofolate synthase, cytoplasmic | P11586 | 102 | 18 | RNA-binding protein Raly | Q9UKM9 | 32 | 9 |
| Peroxidasin homolog | Q92626 | 165 | 17 | Structural maintenance of chromosomes protein 4 | Q9NTJ3 | 147 | 9 |
| 26S protease regulatory subunit 7 | P35998 | 49 | 17 | 60S ribosomal protein L23 | P62829 | 15 | 9 |
| High mobility group protein B1 | Q5T7C4 | 18 | 17 | Elongation factor Tu, mitochondrial | P49411 | 50 | 9 |
| Aspartate--tRNA ligase, cytoplasmic | P14868 | 57 | 17 | Heterogeneous nuclear ribonucleoprotein A1 | P09651 | 39 | 9 |
| 40S ribosomal protein S4, X isoform | P62701 | 30 | 16 | Plasma membrane calcium-transporting ATPase 1 | P20020 | 139 | 9 |
| Ribosome-binding protein 1 | Q9P2E9 | 152 | 16 | 60S ribosomal protein L7 | P18124 | 29 | 9 |
| 40S ribosomal protein S3 | P23396 | 27 | 16 | Eukaryotic translation initiation factor 2 subunit 1 | P05198 | 36 | 9 |
| 6-phosphofructokinase type C | Q01813 | 86 | 16 | Prolow-density lipoprotein receptor-related protein 1 | Q07954 | 505 | 9 |
| Integrin beta-1 | P05556 | 88 | 16 | Glutamine--tRNA ligase | P47897 | 88 | 9 |
| Spectrin alpha chain, brain | Q13813 | 285 | 15 | HLA class I histocompatibility antigen, A-2 alpha chain | P01892 | 41 | 9 |
| 26S proteasome non-ATPase regulatory subunit 11 | O00231 | 47 | 15 | Brain acid soluble protein 1 | P80723 | 23 | 9 |
| 40S ribosomal protein S3a | P61247 | 30 | 15 | Cofilin-1 | P23528 | 19 | 9 |
| Laminin subunit beta-2 | P55268 | 196 | 15 | Triosephosphate isomerase | P60174 | 31 | 9 |
| Heterogeneous nuclear ribonucleoprotein M | P52272 | 78 | 14 | 60S ribosomal protein L6 | Q02878 | 33 | 8 |
| DNA topoisomerase 2-alpha | P11388 | 174 | 14 | Acetyl-CoA carboxylase 1 | Q13085 | 266 | 8 |
| Peroxiredoxin-1 | Q06830 | 22 | 14 | Histone H4 | P62805 | 11 | 8 |
| 26S proteasome non-ATPase regulatory subunit 1 | Q99460 | 106 | 14 | Tropomyosin 3 | Q5VU58 | 29 | 8 |
| Arginine--tRNA ligase, cytoplasmic | P54136 | 75 | 14 | ATP synthase subunit beta, mitochondrial | P06576 | 57 | 8 |
| Laminin subunit alpha-5 | O15230 | 400 | 14 | Disco-interacting protein 2 homolog B | Q9P265 | 171 | 8 |
| 26S protease regulatory subunit 6A | P17980 | 49 | 14 | Lactadherin | Q08431 | 43 | 8 |
| Inosine-5'-monophosphate dehydrogenase 2 | P12268 | 56 | 14 | Ras-related protein Rab-13 | P51153 | 23 | 8 |
| RPL14 protein | Q6IPH7 | 24 | 8 | 60S ribosomal protein L13 | P26373 | 24 | 7 |
| Stress-induced-phosphoprotein 1 | P31948 | 63 | 8 | Kinectin | Q86UP2 | 156 | 7 |
| 26S proteasome non-ATPase regulatory subunit 12 | O00232 | 53 | 8 | Protein S100-A6 | P06703 | 10 | 7 |
| 40S ribosomal protein S9 | P46781 | 23 | 8 | Sorbitol dehydrogenase | Q00796 | 38 | 7 |
| 60S ribosomal protein L3 | P39023 | 46 | 8 | Chloride intracellular channel protein 4 | Q9Y696 | 29 | 7 |
| 60S ribosomal protein L5 | P46777 | 34 | 8 | Eukaryotic translation initiation factor 3 subunit E | P60228 | 52 | 7 |
| Fermitin family homolog 3 | Q86UX7 | 76 | 8 | Guanine nucleotide-binding protein G(I)/G(S)/G(T) subunit beta-2 | P62879 | 37 | 7 |
| Galphai2 protein | Q6B6N3 | 42 | 8 | Integrin alpha-3 | P26006 | 117 | 7 |
| Histone H2B type 2-F | Q5QNW6 | 14 | 8 | Malate dehydrogenase | Q6FHZ0 | 36 | 7 |
| Integrin alpha-6 | P23229 | 127 | 8 | Plasminogen activator inhibitor 1 | P05121 | 45 | 7 |
| Sequestosome-1 | Q13501 | 48 | 8 | Proteasome subunit beta type-2 | P49721 | 23 | 7 |
| Serine protease 23 | O95084 | 43 | 8 | Putative pre-mRNA-splicing factor ATP-dependent RNA helicase DHX15 | O43143 | 91 | 7 |
| Transforming protein RhoA | P61586 | 22 | 8 | Ras-related protein Rap-2b | P61225 | 21 | 7 |
| 26S protease regulatory subunit 6B | P43686 | 47 | 8 | 60S acidic ribosomal protein P2 | P05387 | 12 | 7 |
| Asparagine--tRNA ligase, cytoplasmic | O43776 | 63 | 8 | Heterogeneous nuclear ribonucleoprotein K | P61978 | 51 | 7 |
| Coatomer subunit beta | P53618 | 107 | 8 | Hsp90 co-chaperone Cdc37 | Q16543 | 44 | 7 |
| Ferritin light chain | P02792 | 20 | 8 | Myosin light polypeptide 6 | P60660 | 17 | 7 |
| Heterogeneous nuclear ribonucleoprotein D0 | Q14103 | 38 | 8 | Pre-mRNA-processing factor 19 | Q9UMS4 | 55 | 7 |
| Neutral alpha-glucosidase AB | Q14697 | 107 | 8 | Proteasome subunit beta type-1 | P20618 | 26 | 7 |
| Neutral amino acid transporter B(0) | Q15758 | 57 | 8 | Putative deoxyribose-phosphate aldolase | Q9Y315 | 35 | 7 |
| Poly(rC)-binding protein 1 | Q15365 | 37 | 8 | Stress-70 protein, mitochondrial | P38646 | 74 | 7 |
| Proliferation-associated protein 2G4 | Q9UQ80 | 44 | 8 | 40S ribosomal protein S17-like | P0CW22 | 16 | 6 |
| Proteasome subunit alpha type-3 | P25788 | 28 | 8 | 40S ribosomal protein S19 | P39019 | 16 | 6 |
| Transferrin receptor protein 1 | P02786 | 85 | 8 | 60S ribosomal protein L13a | P40429 | 24 | 6 |
| Activated RNA polymerase II transcriptional coactivator p15 | P53999 | 14 | 8 | 60S ribosomal protein L18 | F8VWC5 | 18 | 6 |
| Aminoacyl tRNA synthase complex-interacting multifunctional protein 2 | Q13155 | 35 | 8 | 60S ribosomal protein L8 | P62917 | 28 | 6 |
| LAMA4 protein | Q5D044 | 202 | 8 | 60S ribosomal protein L9 | P32969 | 22 | 6 |
| Proteasome subunit alpha type-4 | P25789 | 29 | 8 | BAG family molecular chaperone regulator 2 | O95816 | 24 | 6 |
| 60S ribosomal protein L17 | P18621 | 21 | 7 | Calnexin | P27824 | 68 | 6 |
| 60S ribosomal protein L26 | P61254 | 17 | 7 | Carboxypeptidase D | O75976 | 153 | 6 |
| 60S ribosomal protein L28 | P46779 | 16 | 7 | DnaJ homolog subfamily C member 13 | O75165 | 254 | 6 |
| Citron | Q2M5E1 | 237 | 7 | Kinase D-interacting substrate of 220 | Q9ULH0 | 197 | 6 |
| Histone cluster 1, H1e | Q4VB24 | 22 | 7 | Neurogenic locus notch homolog protein 2 | Q04721 | 265 | 6 |
| Myosin-10 | P35580 | 229 | 7 | Ras-related protein Rab-5C | P51148 | 23 | 6 |
| Phenylalanine--tRNA ligase beta subunit | Q9NSD9 | 66 | 7 | Receptor-type tyrosine-protein phosphatase F | P10586 | 213 | 6 |
| Ras-related protein Rap-1b | P61224 | 21 | 7 | Serine/arginine-rich splicing factor 3 | P84103 | 19 | 6 |
| Sister chromatid cohesion protein PDS5 homolog A | Q29RF7 | 151 | 7 | Slit homolog 2 protein | O94813 | 170 | 6 |
| 40S ribosomal protein S8 | P62241 | 24 | 7 | Unconventional myosin-Ic | O00159 | 122 | 6 |
| 40S ribosomal protein SA | P08865 | 33 | 7 |  |  |  |  |
|  |  |  |  |  |  |  |  |
|  |  |  |  |  |  |  |  |
|  |  |  |  |  |  |  |  |

**Supplementary Table S3D: Hige expression in HG-NV of MDA-MB-231 cells with proteomics analysis by HPLC-MS**

| **Identified Proteins** | **Access #** | **MW**  **(kDa)** | **Counts** | **Identified Proteins** | **Access #** | **MW**  **(kDa)** | **Counts** |
| --- | --- | --- | --- | --- | --- | --- | --- |
| Serum albumin | P02769 | 69 | 74 | Alpha-fetoprotein | P02771 | 69 | 9 |
| Vimentin | P08670 | 54 | 47 | C99 | B4DII8 | 85 | 9 |
| Heat shock cognate 71 kDa protein | P11142 | 71 | 35 | COP9 signalosome complex subunit 3 | Q9UNS2 | 48 | 9 |
| Keratin, type II cytoskeletal 1 | P04264 | 66 | 30 | Glycogen phosphorylase, brain form | P11216 | 97 | 9 |
| Keratin, type II cytoskeletal 6B | P04259 | 60 | 25 | Malate dehydrogenase, cytoplasmic | P40925 | 36 | 9 |
| Keratin, type I cytoskeletal 16 | P08779 | 51 | 23 | Periostin | Q15063 | 93 | 9 |
| Importin-5 | O00410 | 124 | 22 | 26S proteasome non-ATPase regulatory subunit 14 | O00487 | 35 | 9 |
| Actinin alpha 1 isoform b | Q1HE25 | 106 | 21 | Acetyl-CoA acetyltransferase, cytosolic | Q9BWD1 | 41 | 9 |
| Keratin, type I cytoskeletal 10 | P13645 | 59 | 18 | Cathepsin D | P07339 | 45 | 9 |
| Fructose-bisphosphate aldolase A | P04075 | 39 | 16 | Cytoplasmic dynein 1 light intermediate chain 1 | Q9Y6G9 | 57 | 9 |
| Keratin, type I cytoskeletal 9 | P35527 | 62 | 16 | Cytosolic non-specific dipeptidase | Q96KP4 | 53 | 9 |
| Exportin-2 | P55060 | 110 | 16 | Fascin | Q16658 | 55 | 9 |
| Lysyl oxidase homolog 2 | Q9Y4K0 | 87 | 15 | Nuclease-sensitive element-binding protein 1 | P67809 | 36 | 9 |
| X-ray repair cross-complementing  protein 5 | P13010 | 83 | 15 | Peroxiredoxin-6 | P30041 | 25 | 9 |
| Pentraxin-related protein PTX3 | P26022 | 42 | 15 | Purine nucleoside phosphorylase | P00491 | 32 | 9 |
| Adenylyl cyclase-associated protein 1 | Q01518 | 52 | 15 | Replication protein A 70 kDa DNA-binding subunit | P27694 | 68 | 9 |
| Amyloid-like protein 2 | Q06481 | 87 | 14 | Vacuolar protein sorting-associated protein 28 homolog | Q9UK41 | 25 | 9 |
| Alpha-actinin-4 | O43707 | 105 | 14 | 4-trimethylaminobutyraldehyde dehydrogenase | P49189 | 54 | 8 |
| Keratin, type II cytoskeletal 2 epidermal | P35908 | 65 | 14 | ADAM metallopeptidase domain 30 | Q8TBZ7 | 89 | 8 |
| Interleukin enhancer-binding factor 3 | Q12906 | 95 | 13 | Heat shock 70 kDa protein 13 | P48723 | 52 | 8 |
| Serine/threonine-protein phosphatase 2A 65 kDa regulatory subunit A alpha isoform | P30153 | 65 | 13 | Histidine--tRNA ligase, cytoplasmic | P12081 | 57 | 8 |
| 60S acidic ribosomal protein P0 | P05388 | 34 | 13 | Keratin, type II cytoskeletal 6A | P02538 | 60 | 8 |
| ATP-dependent RNA helicase A | Q08211 | 141 | 13 | Lactotransferrin | P02788 | 78 | 8 |
| Interstitial collagenase | P03956 | 54 | 11 | Phosphoglucomutase-1 | P36871 | 61 | 8 |
| Keratin, type I cytoskeletal 14 | P02533 | 52 | 11 | Probable serine carboxypeptidase CPVL | Q9H3G5 | 54 | 8 |
| Proteasome activator complex subunit 1 | Q06323 | 29 | 11 | Renin receptor | O75787 | 39 | 8 |
| Synaptic vesicle membrane protein VAT- 1 homolog | Q99536 | 42 | 11 | Zinc finger FYVE domain-containing protein 1 | Q9HBF4 | 87 | 8 |
| Splicing factor 3B subunit 3 | Q15393 | 136 | 11 | Zinc finger protein RFP | P14373 | 58 | 8 |
| WD repeat-containing protein 1 | O75083 | 66 | 11 | 60S acidic ribosomal protein P1 | P05386 | 12 | 8 |
| Vitamin D-binding protein | P02774 | 53 | 10 | A-kinase anchor protein 13 | H7BYL5 | 308 | 8 |
| Keratin, type II cytoskeletal 5 | P13647 | 62 | 10 | Fumarate hydratase, mitochondrial | P07954 | 55 | 8 |
| Keratin, type II cytoskeletal 8 | P05787 | 54 | 10 | Importin-9 | Q96P70 | 116 | 8 |
| Transformation-related protein 14 | Q597H1 | 43 | 10 | Protein SET | Q01105 | 33 | 8 |
| Heat shock 70 kDa protein 4 | P34932 | 94 | 10 | Serglycin | P10124 | 18 | 8 |
| Heat shock protein 105 kDa | Q92598 | 97 | 10 | Titin | Q8WZ42 | 3816 | 8 |
| Heat shock protein 75 kDa, mitochondrial | Q12931 | 80 | 9 | Urokinase-type plasminogen activator | P00749 | 49 | 8 |
| Proactivator polypeptide | P07602 | 58 | 9 | Very long-chain specific acyl-CoA dehydrogenase,  mitochondrial | P49748 | 70 | 8 |
| von Willebrand factor | P04275 | 309 | 9 | Voltage-dependent R-type calcium channel subunit alpha-1E | Q15878 | 262 | 8 |
