# Supplementary material for: Isolation, identification, and characterization of novel nanovesicles
Source: Oncotarget. 2016 May 12;7(27):41346–62. doi: 10.18632/oncotarget.9325 (PMC5173064; doi:10.18632/oncotarget.9325)
Supplement: Supplementary file 4 [file oncotarget-07-41346-s004.docx]

# Supplementary Table S2A: High-level RNA detected in exosome of 4T1

| **Identified Proteins** |  | **Counts log2-** | | | **Identified Proteins** |  | **Counts** | | **log2-** |
| --- | --- | --- | --- | --- | --- | --- | --- | --- | --- |
|  | **exo** | **HG-NV fold chang** | | |  | **exo** | **HG-NV** | | **fold chang** |
| mitochondrially encoded 16S rRNA | 8647 |  | 53 | 7.3 | potassium channel tetramerisation domain containing 10 | 670 |  | 29 | 4.5 |
| mitochondrially encoded 12S rRNA | 11722 | | 87 | 7.1 | ribosomal protein L18A | 795 |  | 35 | 4.5 |
| RNA, Y3 small cytoplasmic (asso Ro  protein) | 1882 |  | 16 | 6.8 | zinc finger, CCHC domain containing 24 | 330 |  | 14 | 4.5 |
| ribosomal protein L14 | 518 |  | 6 | 6.2 | ubiquitin-conjugating enzyme E2D 3 | 109 |  | 4 | 4.5 |
| ribosomal protein L23 | 409 |  | 5 | 6.1 | tyrosine 3-monooxygenase/tryptophan  5-monooxygenase activation protein, zeta polypeptide | 241 |  | 10 | 4.5 |
| RNA, Y1 small cytoplasmic, Ro-  associated | 1451 |  | 21 | 6.0 | nudix (nucleoside diphosphate linked  moiety X)-type motif 4 | 194 |  | 8 | 4.4 |
| RNA, 7SK, nuclear | 2568 |  | 42 | 5.9 | poly(A) binding protein, cytoplasmic 1 | 3178 |  | 146 | 4.4 |
| predicted gene 15564 | 6086 |  | 102 | 5.9 | tubulin, beta 5 class I | 427 |  | 19 | 4.4 |
| mitochondrially encoded cytochrome b | 696 |  | 11 | 5.9 | predicted gene 9625 | 127 |  | 5 | 4.4 |
| predicted gene, 22973 | 376 |  | 6 | 5.8 | ribosomal protein S16 | 126 |  | 5 | 4.4 |
| golgi SNAP receptor complex member 2 | 688 |  | 12 | 5.7 | histone cluster 1, H1e | 336 |  | 15 | 4.4 |
| mitochondrially encoded cytochrome c oxidase I | 785 |  | 14 | 5.7 | U2AF homology motif (UHM) kinase 1 | 483 |  | 22 | 4.4 |
| ribosomal protein S20 | 393 |  | 7 | 5.6 | H3 histone, family 3B | 417 |  | 19 | 4.4 |
| ribosomal protein S11 | 1198 |  | 24 | 5.6 | sestrin 3 | 145 |  | 6 | 4.4 |
| guanosine diphosphate dissociation inhibitor 2 | 204 |  | 4 | 5.4 | methylthioadenosine phosphorylase | 145 |  | 6 | 4.4 |
| solute carrier family 25, member 4 | 309 |  | 7 | 5.3 | glia maturation factor, beta | 144 |  | 6 | 4.4 |
| ferritin light chain 1 | 2905 |  | 74 | 5.3 | ribosomal protein L30 | 304 |  | 14 | 4.3 |
| predicted gene 15772 | 881 |  | 22 | 5.3 | transcription factor 20 | 970 |  | 47 | 4.3 |
| stearoyl-Coenzyme A desaturase 2 | 612 |  | 15 | 5.3 | metastasis associated lung adenocarcinoma transcript 1 | 342 |  | 16 | 4.3 |
| actin, beta | 686 |  | 18 | 5.2 | pantothenate kinase 3 | 180 |  | 8 | 4.3 |
| ribosomal protein S26 | 276 |  | 7 | 5.1 | S100 calcium binding protein A6 (calcyclin) | 159 |  | 7 | 4.3 |
| lectin, galactose binding, soluble 1 | 687 |  | 19 | 5.1 | ribosomal protein S21 | 199 |  | 9 | 4.3 |
| spindlin 1 | 205 |  | 5 | 5.1 | predicted gene, 26191 | 458 |  | 22 | 4.3 |
| ribosomal protein L35 | 230 |  | 6 | 5.0 | leucine rich repeat containing 58 | 157 |  | 7 | 4.3 |
| eukaryotic translation initiation factor 3, subunit A | 719 |  | 21 | 5.0 | mitochondrially encoded NADH dehydrogenase 5 | 662 |  | 33 | 4.3 |
| ferritin heavy chain 1 | 2434 |  | 74 | 5.0 | ribosomal protein L32 | 308 |  | 15 | 4.3 |
| mitochondrially encoded NADH dehydrogenase 1 | 410 |  | 12 | 5.0 | ribosomal protein S27A | 286 |  | 14 | 4.3 |
| AHNAK nucleoprotein (desmoyokin) | 1688 |  | 53 | 5.0 | trafficking protein, kinesin binding 2 | 668 |  | 34 | 4.3 |
| nucleolin | 3301 |  | 105 | 5.0 | nuclear fragile X mental retardation  protein interacting protein 2 | 94 |  | 4 | 4.2 |
| predicted pseudogene 8730 | 464 |  | 14 | 5.0 | cytochrome b5 reductase 3 | 529 |  | 27 | 4.2 |
| eukaryotic translation initiation factor 4E binding protein 2 | 305 |  | 9 | 4.9 | predicted gene, 22405 | 112 |  | 5 | 4.2 |
| ribosomal protein S7 | 458 |  | 14 | 4.9 | transformation related protein 53 inducible nuclear protein 2 | 260 |  | 13 | 4.2 |
| nucleophosmin 1 | 1600 |  | 52 | 4.9 | profilin 1 | 184 |  | 9 | 4.2 |
| Finkel-Biskis-Reilly murine sarcoma virus ubiquitously expressed | 149 |  | 4 | 4.9 | RNA binding motif protein 3 | 91 |  | 4 | 4.2 |
| ribosomal protein L13A | 415 |  | 13 | 4.9 | glutathione peroxidase 4 | 91 |  | 4 | 4.2 |

| DnaJ (Hsp40) homolog, subfamily A, member 2 | 198 | 6 | 4.8 | ribosomal protein, large, P0 | 1157 | 62 | 4.2 |
| --- | --- | --- | --- | --- | --- | --- | --- |
| ribosomal protein L28 | 396 | 13 | 4.8 | charged multivesicular body protein 3 | 127 | 6 | 4.2 |
| aldolase A, fructose-bisphosphate | 509 | 17 | 4.8 | ribosomal protein L17 | 180 | 9 | 4.2 |
| guanine nucleotide binding protein (G protein), beta polypeptide 2 like 1 | 422 | 14 | 4.8 | fibroblast growth factor receptor substrate  2 | 161 | 8 | 4.2 |
| histone cluster 1, H4d | 251 | 8 | 4.8 | heat shock protein 90 alpha (cytosolic),  class B member 1 | 1335 | 74 | 4.2 |
| mitochondrially encoded NADH dehydrogenase 2 | 334 | 11 | 4.8 | ribosomal protein L12 | 367 | 20 | 4.1 |
| ribosomal protein S9 | 466 | 16 | 4.8 | guanine nucleotide binding protein, alpha 13 | 259 | 14 | 4.1 |
| Rho GTPase activating protein 11A | 567 | 20 | 4.8 | prothymosin alpha | 120 | 6 | 4.1 |
| ribosomal protein L36 | 214 | 7 | 4.7 | ornithine decarboxylase, structural 1 | 1218 | 70 | 4.1 |
| neuroepithelial cell transforming gene 1 | 373 | 13 | 4.7 | heat shock protein 9 | 205 | 11 | 4.1 |
| ribosomal protein S3A1 | 665 | 24 | 4.7 | ribosomal protein L8 | 906 | 52 | 4.1 |
| ribosomal protein S23 | 158 | 5 | 4.7 | platelet-activating factor acetylhydrolase, isoform 1b, subunit 2 | 187 | 10 | 4.1 |
| histone cluster 1, H2ak | 130 | 4 | 4.7 | ribosomal protein L13 | 611 | 35 | 4.1 |
| eukaryotic translation elongation factor 1 alpha 1 | 3271 | 124 | 4.7 | ribosomal protein S14 | 135 | 7 | 4.1 |
| mitochondrially encoded NADH dehydrogenase 4 | 225 | 8 | 4.7 | growth arrest specific 5 | 556 | 32 | 4.1 |
| ribosomal protein L37 | 249 | 9 | 4.6 | pyruvate kinase, muscle | 487 | 28 | 4.1 |
| ribonuclease P RNA component H1 | 8824 | 354 | 4.6 | keratin 18 | 150 | 8 | 4.1 |
| N(alpha)-acetyltransferase 50, NatE catalytic subunit | 148 | 5 | 4.6 | family with sequence similarity 168, member B | 753 | 44 | 4.1 |
| ribosomal protein S6 | 615 | 24 | 4.6 | Rho GDP dissociation inhibitor (GDI) alpha | 233 | 13 | 4.1 |
| ribosomal protein L4 | 658 | 26 | 4.6 | lactate dehydrogenase A | 248 | 14 | 4.1 |
| acidic (leucine-rich) nuclear phosphoprotein 32 family, member B | 437 | 17 | 4.6 | eukaryotic translation initiation factor 3, subunit E | 115 | 6 | 4.1 |
| ribosomal protein L19 | 1067 | 43 | 4.6 | cold shock domain containing E1, RNA binding | 506 | 30 | 4.0 |
| eukaryotic translation initiation factor 4, gamma 2 | 377 | 15 | 4.6 | transforming growth factor, beta receptor I | 178 | 10 | 4.0 |
| ribosomal protein L41 | 351 | 14 | 4.6 | ribosomal protein L38 | 178 | 10 | 4.0 |
| LSM12 homolog (S. cerevisiae) | 139 | 5 | 4.5 | microtubule-associated protein, RP/EB family, member 1 | 551 | 33 | 4.0 |
| PRELI domain containing 1 | 278 | 11 | 4.5 | ring finger and CCCH-type zinc finger  domains 2 | 355 | 21 | 4.0 |
| chromobox 5 | 763 | 32 | 4.5 | actin, gamma, cytoplasmic 1 | 257 | 15 | 4.0 |
| ribosomal protein SA | 1285 | 55 | 4.5 | nuclear factor I/X | 384 | 23 | 4.0 |
| eukaryotic translation elongation factor 2 | 1493 | 65 | 4.5 | ribosomal protein S10 | 79 | 4 | 4.0 |
| vesicle amine transport protein 1 homolog (T californica) | 134 | 5 | 4.5 |  |  |  |  |

**Supplementary Table S2B: High-level RNA detected in HG-NV of 4T1**

| **Identified Proteins** | **Counts** | **log2-** | | **Identified Proteins** | **Counts** |  | **log2-** |
| --- | --- | --- | --- | --- | --- | --- | --- |
|  | **exo** | **HG-NV fold chang** | |  | **exo** | **HG- NV** | **fold chang** |
| cDNA sequence BC018473 | 0 | 31 | –5.0 | cytochrome P450, family 4, subfamily  a, polypeptide 29,  pseudogene 1 | 0 | 9 | -3.3 |
| reticulon 4 receptor | 0 | 24 | –4.6 | predicted gene 14893 | 0 | 9 | –3.3 |
| surfactant associated 2 | 0 | 21 | –4.5 | predicted gene 14108 | 0 | 9 | –3.3 |
| predicted gene, 21786 | 0 | 20 | –4.4 | predicted gene 15666 | 0 | 9 | –3.3 |
| olfactory receptor 430 | 0 | 17 | –4.2 | RIKEN cDNA E130120K24 gene | 0 | 9 | –3.3 |
| predicted gene 7672 | 0 | 16 | –4.1 | predicted gene 13716 | 0 | 9 | –3.3 |
| interferon induced transmembrane protein 1 | 0 | 15 | –4.0 | predicted gene, 24500 | 0 | 9 | –3.3 |
| expressed sequence BB014433 | 0 | 15 | –4.0 | matrin 3, pseudogene 2 | 0 | 9 | –3.3 |
| predicted gene 9443 | 0 | 15 | –4.0 | predicted gene 8356 | 0 | 9 | –3.3 |
| vomeronasal 1 receptor 212 | 0 | 14 | –3.9 | histocompatibility 2, Q  region locus 2 | 0 | 9 | –3.3 |
| predicted gene 13031 | 0 | 13 | –3.8 | predicted gene 20447 | 0 | 9 | –3.3 |
| T cell receptor alpha variable 7D–3 | 0 | 13 | –3.8 | serine/cysteine peptidase inhibitor, clade B (ovalbumin), member 10 | 0 | 9 | –3.3 |
| predicted gene 29539 | 0 | 13 | –3.8 | predicted gene, 18006 | 0 | 9 | –3.3 |
| Indian hedgehog | 0 | 12 | –3.7 | predicted gene, 27784 | 0 | 9 | –3.3 |
| catechol-O-methyltransferase domain containing 1 | 0 | 12 | –3.7 | TEC RP24-329M13.1 | 0 | 9 | –3.3 |
| predicted gene, 22061 | 0 | 12 | –3.7 | predicted gene 5973 | 2 | 28 | –3.3 |
| predicted gene, 23247 | 0 | 12 | –3.7 | TEC RP23-184I13.2 | 2 | 28 | –3.3 |
| predicted gene 8225 | 0 | 12 | –3.7 | cysteine-rich secretory protein 4 | 1 | 18 | –3.2 |
| CCAAT/enhancer binding protein | 0 | 12 | –3.7 | predicted pseudogene 5540 | 1 | 18 | –3.2 |
| histocompatibility 2, blastocyst | 0 | 12 | –3.7 | predicted gene 13446 | 1 | 18 | –3.2 |
| olfactory receptor 1156 | 0 | 12 | –3.7 | predicted gene, 26617 | 1 | 18 | –3.2 |
| immunoglobulin kappa variable 3–10 | 0 | 12 | –3.7 | RIKEN cDNA  4930455D15 gene | 1 | 18 | –3.2 |
| predicted gene 11931 | 0 | 12 | –3.7 | ankyrin repeat domain 33 | 2 | 27 | –3.2 |
| predicted gene 12869 | 0 | 12 | –3.7 | predicted gene 15784 | 2 | 27 | –3.2 |
| predicted gene 13434 | 0 | 12 | –3.7 | lymphocyte antigen 6 complex, locus G6E | 0 | 8 | –3.2 |
| predicted gene, 26752 | 0 | 12 | –3.7 | interleukin 1 family,  member 8 | 0 | 8 | –3.2 |
| predicted gene 29150 | 0 | 12 | –3.7 | late cornified envelope-  like proline-rich 1 | 0 | 8 | –3.2 |
| predicted pseudogene 336 | 0 | 12 | –3.7 | predicted pseudogene 5578 | 0 | 8 | –3.2 |
| predicted gene 12010 | 1 | 24 | –3.6 | olfactory receptor 981 | 1 | 17 | –3.2 |
| predicted pseudogene 8818 | 0 | 11 | –3.6 | predicted gene 5065 | 0 | 8 | –3.2 |
| predicted gene 13771 | 0 | 11 | –3.6 | sorting nexin 32 | 1 | 17 | –3.2 |
| predicted gene 2381 | 0 | 11 | –3.6 | protease, serine 34 | 1 | 17 | –3.2 |
| predicted gene 20611 | 0 | 11 | –3.6 | serine/cysteine peptidase inhibitor, clade B (ovalbumin), member 3D | 0 | 8 | –3.2 |
| predicted gene 5435 | 0 | 11 | –3.6 | olfactory receptor 539 | 0 | 8 | –3.2 |

| predicted gene 28351 | 0 | 11 | –3.6 | complement factor D (adipsin) | 0 | 8 | –3.2 |
| --- | --- | --- | --- | --- | --- | --- | --- |
| predicted gene 8531 | 0 | 11 | –3.6 | RIKEN cDNA  4930431F12 gene | 0 | 8 | –3.2 |
| predicted gene 29441 | 0 | 11 | –3.6 | olfactory receptor 319 | 0 | 8 | –3.2 |
| TEC RP23-420P19.1 | 0 | 11 | –3.6 | hemoglobin alpha, adult chain 1 | 0 | 8 | –3.2 |
| secretoglobin, family 1B, member 20 | 1 | 22 | –3.5 | microRNA 489 | 0 | 8 | –3.2 |
| T–box 1 | 0 | 10 | –3.5 | predicted gene 16020 | 0 | 8 | –3.2 |
| olfactory receptor 367, pseudogene | 0 | 10 | –3.5 | NEDD4 binding protein 2, opposite strand | 0 | 8 | –3.2 |
| Sec61 beta subunit | 1 | 21 | –3.5 | predicted gene 10518 | 0 | 8 | –3.2 |
| predicted gene, 26228 | 0 | 10 | –3.5 | predicted gene, 25614 | 0 | 8 | –3.2 |
| ankyrin repeat domain 63 | 2 | 32 | –3.5 | immunoglobulin kappa variable 12–47 | 0 | 8 | –3.2 |
| predicted gene 13983 | 0 | 10 | –3.5 | predicted gene 11434 | 0 | 8 | –3.2 |
| nuclear encoded rRNA 5S 34 | 0 | 10 | –3.5 | RIKEN cDNA  2210409E12 gene | 0 | 8 | –3.2 |
| predicted gene 12590 | 0 | 10 | –3.5 | guanine nucleotide binding protein (G protein), gamma 2  subunit, pseudogene 1 | 0 | 8 | –3.2 |
| predicted gene 14262 | 0 | 10 | –3.5 | predicted gene 8475 | 0 | 8 | –3.2 |
| RIKEN cDNA 4930515B02 gene | 0 | 10 | –3.5 | predicted gene 12191 | 0 | 8 | –3.2 |
| RIKEN cDNA 1700073E17 gene | 0 | 10 | –3.5 | predicted gene 14805 | 0 | 8 | –3.2 |
| predicted gene 15860 | 0 | 10 | –3.5 | predicted gene 15159 | 0 | 8 | –3.2 |
| predicted gene 15775 | 0 | 10 | –3.5 | predicted gene 15812 | 0 | 8 | –3.2 |
| predicted gene, 25958 | 0 | 10 | –3.5 | predicted gene 16064 | 0 | 8 | –3.2 |
| predicted gene 12626 | 0 | 10 | –3.5 | RIKEN cDNA 1700123O12 gene | 0 | 8 | –3.2 |
| TEC RP23-272A7.1 | 0 | 10 | –3.5 | cDNA sequence  BC039966 | 1 | 17 | –3.2 |
| processed_pseudogene RP23- 215I1.2 | 0 | 10 | –3.5 | predicted gene, 25603 | 0 | 8 | –3.2 |
| beta-1,3-glucuronyltransferase 2 | 1 | 20 | –3.4 | predicted gene 6397 | 0 | 8 | –3.2 |
| homeobox A11 | 1 | 19 | –3.3 | predicted gene 8428 | 1 | 17 | –3.2 |
| RIKEN cDNA C130073F10 gene | 0 | 9 | –3.3 | predicted gene, 22002 | 0 | 8 | –3.2 |
| olfactory receptor 250 | 0 | 9 | –3.3 | predicted gene, 21847 | 0 | 8 | –3.2 |
| microRNA 369 | 0 | 9 | –3.3 | vomeronasal 1 receptor  30 | 0 | 8 | –3.2 |
| defensin beta 43 | 0 | 9 | –3.3 | interferon alpha 15 | 0 | 8 | –3.2 |
| predicted gene 11553 | 0 | 9 | –3.3 | predicted gene, 16907 | 4 | 44 | –3.2 |
| predicted gene 15381 | 0 | 9 | –3.3 | predicted gene, 17800 | 0 | 8 | –3.2 |
| predicted gene 12386 | 0 | 9 | –3.3 | predicted gene, 26980 | 0 | 8 | –3.2 |
| predicted gene 15660 | 0 | 9 | –3.3 | predicted gene 29253 | 0 | 8 | –3.2 |
| predicted gene 5319 | 0 | 9 | –3.3 | predicted gene 7114 | 0 | 8 | –3.2 |
| TEC RP23-259O15.2 | 0 | 8 | –3.2 | cholinergic receptor, muscarinic 4 | 2 | 23 | –3.0 |
| TEC RP23-141H24.1 | 0 | 8 | –3.2 | predicted gene 12221 | 0 | 7 | –3.0 |
| TEC RP23-293F4.2 | 0 | 8 | –3.2 | olfactory receptor 1388 | 0 | 7 | –3.0 |
| processed_pseudogene RP23-  464I9.1 | 0 | 8 | –3.2 | fibroblast growth factor  binding protein 1 | 0 | 7 | –3.0 |
| leucine rich repeat containing 32 | 2 | 25 | –3.1 | interferon beta 1,  fibroblast | 0 | 7 | –3.0 |

| predicted gene 4745 | 1 | 16 | –3.1 | small proline-rich protein 2B | 0 | 7 | –3.0 |
| --- | --- | --- | --- | --- | --- | --- | --- |
| zona pellucida like domain containing 1 | 1 | 16 | –3.1 | olfactory receptor 1356 | 0 | 7 | –3.0 |
| T cell receptor beta, variable 16 | 1 | 16 | –3.1 | WAP four-disulfide core  domain 21 | 0 | 7 | –3.0 |
| RIKEN cDNA 2210017G18 gene | 1 | 16 | –3.1 | activator of yeast meiotic promoters 1 | 0 | 7 | –3.0 |
| predicted gene, 27043 | 1 | 16 | –3.1 | RIKEN cDNA M5C1000I18 gene | 1 | 15 | –3.0 |
| TEC RP24-111F24.1 | 1 | 16 | –3.1 | prolactin family 3, subfamily d, member 1 | 0 | 7 | –3.0 |
| icos ligand | 4 | 41 | –3.1 | vomeronasal 1 receptor  232 | 0 | 7 | –3.0 |
| gasdermin C-like 1 | 2 | 24 | –3.1 | transmembrane protein 235 | 1 | 15 | –3.0 |
| predicted gene 21975 | 2 | 24 | –3.1 | formyl peptide receptor, related sequence 6 | 0 | 7 | –3.0 |
| RIKEN cDNA E530011L22 gene | 2 | 24 | –3.1 | zinc finger protein 456 | 0 | 7 | –3.0 |
| glycine receptor, alpha 4 subunit | 0 | 7 | –3.0 | serine (or cysteine) peptidase inhibitor, clade B, member 1c | 0 | 7 | –3.0 |
| homeobox C10 | 1 | 15 | –3.0 | predicted gene 12838 | 0 | 7 | –3.0 |
| glycoprotein m6a | 0 | 7 | –3.0 | predicted gene 12006 | 0 | 7 | –3.0 |
